# Spatiotemporal patterns of locus coeruleus integrity predict cortical tau and cognition

**DOI:** 10.1038/s43587-024-00626-y

**Published:** 2024-04-25

**Authors:** Elisenda Bueichekú, Ibai Diez, Chan-Mi Kim, John Alex Becker, Elouise A. Koops, Kenneth Kwong, Kathryn V. Papp, David H. Salat, David A. Bennett, Dorene M. Rentz, Reisa A. Sperling, Keith A. Johnson, Jorge Sepulcre, Heidi I. L. Jacobs

**Affiliations:** 1 Gordon Center for Medical Imaging, Department of Radiology, Massachusetts General Hospital, Boston, MA USA; 2 Harvard Medical School, Boston, MA USA; 3 The Athinoula A. Martinos Center for Biomedical Imaging, Department of Radiology, Massachusetts General Hospital, Boston, MA USA; 4 Center for Alzheimer Research and Treatment, Department of Neurology, Brigham and Women’s Hospital, Boston, MA USA; 5 Department of Neurology, Massachusetts General Hospital, Boston, MA USA; 6 Neuroimaging Research for Veterans Center, VA Boston Healthcare System, Boston, MA USA; 7 Rush Alzheimer’s Disease Center, Rush University Medical Center, Chicago, IL USA; 8 Department of Neurological Sciences, Rush University Medical Center, Chicago, IL USA; 9 Department of Radiology, Yale PET Center, Yale Medical School, Yale University, New Haven, CT USA; 10 Faculty of Health, Medicine and Life Sciences, School for Mental Health and Neuroscience, Alzheimer Centre Limburg, Maastricht University, Maastricht, Netherlands

**Keywords:** Neuroscience, Predictive markers, Ageing

## Abstract

Autopsy studies indicated that the locus coeruleus (LC) accumulates hyperphosphorylated tau before allocortical regions in Alzheimer’s disease. By combining in vivo longitudinal magnetic resonance imaging measures of LC integrity, tau positron emission tomography imaging and cognition with autopsy data and transcriptomic information, we examined whether LC changes precede allocortical tau deposition and whether specific genetic features underlie LC’s selective vulnerability to tau. We found that LC integrity changes preceded medial temporal lobe tau accumulation, and together these processes were associated with lower cognitive performance. Common gene expression profiles between LC–medial temporal lobe–limbic regions map to biological functions in protein transport regulation. These findings advance our understanding of the spatiotemporal patterns of initial tau spreading from the LC and LC’s selective vulnerability to Alzheimer’s disease pathology. LC integrity measures can be a promising indicator for identifying the time window when individuals are at risk of disease progression and underscore the importance of interventions mitigating initial tau spread.

## Abstract (web summary)

In vivo human neuroimaging shows that locus coeruleus (LC) integrity changes precede medial temporal tau accumulation, and jointly predict future lower cognition in older people at risk for Alzheimer’s disease. A common transcriptomic profile underlies LC’s early vulnerability to tau.

## Main

Alzheimer’s disease (AD) pathophysiology begins decades before cognitive decline is clinically noticeable^1^, making it a priority to investigate early neurobiological mechanisms implicated in its neurodegenerative processes^2^. An early neuropathologic hallmark of AD is the abnormal accumulation of hyperphosphorylated tau protein, which is postulated to progress throughout the brain with a rather predictable topography. As described in Braak staging, hyperphosphorylated tau aggregates accumulate first in the brainstem, including the LC, and then follow a hierarchically organized progression into the entorhinal cortex, to other regions of the allocortex and eventually to the neocortex^3–7^. Whether the LC is the seed of tau spreading to the entorhinal cortex remains a topic of debate, with growing research suggesting that tau spreads from the LC to the medial temporal lobe (MTL) structures^5,6^, but also human histopathology work reporting LC seeding only after the emergence of tau in the entorhinal cortex^8^. As clinical trials are moving to individuals in earlier, asymptomatic stages, resolving the question of whether the LC is the origin of tau pathology and what makes the LC specifically vulnerable to early tau deposition will contribute to more accurately determining the ideal window of opportunity for enrollment in clinical trials.

Our group has developed magnetic resonance imaging (MRI) approaches to evaluate LC integrity in vivo, as reliable detection of tau pathology with positron emission tomography (PET) images of the LC is not yet feasible given its small size relative to the resolution and the off-target binding of tau tracers to neuromelanin, which is also present in the LC. Recently, we reported cross-sectional associations between in vivo tau PET in MTL structures and LC integrity in asymptomatic older individuals. In autopsy data, we found similar strong associations between LC tangle density and allocortical and neocortical tangle density, supporting the conclusion that LC integrity conveys information about tau-related processes in the LC^9^. Later, we described analogous observations in individuals with preclinical autosomal-dominant AD^10^. In autosomal-dominant AD, LC integrity started to decline before the estimated detection ability of allo- and neocortical tau PET and declines in LC integrity correlated with increases in allocortical tau, suggesting a potential sequence consistent with Braak staging. In both sporadic and autosomal-dominant AD, we observed that lower LC integrity and worse memory performance was mediated by initial allocortical tau pathology, suggesting that the progression of pathology to allocortical regions was affecting the process by which the LC–norepinephrine (NE) system modulates cognition and behavior, although there is still no in vivo longitudinal evidence of LC changes preceding allo- and neocortical tau spreading. LC neurons are particularly vulnerable to AD-related pathology and factors proposed to contribute to this vulnerability include poorly myelinated projections fibers to the cortex, increased gene and protein turnover rates or accumulation of environmental toxins^11,12^. Even though these hypotheses have been proposed, the biological processes or mechanisms contributing to specific tau propagation patterns involving the LC remain unknown. In this study, we aimed to examine potential spatiotemporal routes of pathology progression at the individual level, their association with cognitive outcomes, and identify underlying genetic features contributing to their vulnerability to tau. By taking a neurogenetic approach, we will be able to describe the shared genetic background and provide neurobiological hypotheses underlying the specific tau propagation pathways between the LC and cortex in the initial stages of the disease. Following our previous observations and the available autopsy data, we hypothesized that lower LC integrity predicts future accumulation of initial allo- and neocortical tau better than MTL allocortical tau accumulation predicting lower LC integrity. Furthermore, we hypothesized that these specific directional spatiotemporal LC–allocortical tau relationships are associated with poor cognitive performance. Finally, to understand specific biological features underlying the vulnerability of early tau pathways from the LC to the cortex, we used the Allen Human Brain Atlas (AHBA) transcriptome data of protein-coding genes. To evaluate our hypotheses, we combined two-time points of LC MR imaging, tau PET imaging and cognitive data of 77 well-characterized individuals followed for up to 3 years and validated whether the spatiotemporal tau spreading findings are supported by estimated spatial staging of tangle density measures in the LC versus those in initial allocortical regions using neuropathologic data from the Rush Memory and Aging Project (MAP)^13,14^.

## Results

### Demographics of the samples

**Extended Data Fig. 1:**
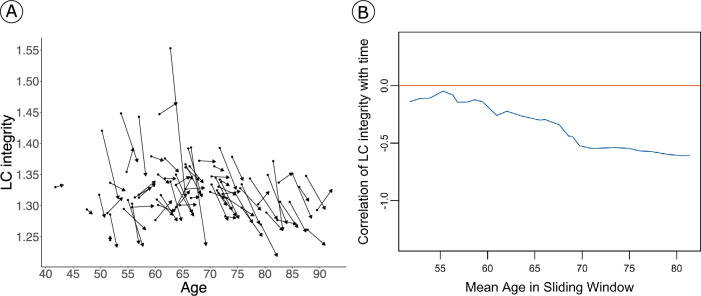
Change in LC integrity signal as a function of age. **a**) Decreases in LC integrity are observed across all ages. Every pair of dots represents one individual (n = 77), and arrows represent the direction of the change. **b**) The sliding window correlation plot also demonstrates that LC integrity decreases over time across all ages (blue line=correlation between LC integrity and time across age (binned by 15 years)).

All participants (*n* = 77, 50 females (65%)) had two 3T MRIs, including our dedicated LC sequence and PET sessions (baseline, age range 41.75 to 89.50 years; follow-up age range, 43.25 to 92.25 years; Supplementary Table 1a). Using our previously established methods^9^, we quantified LC intensity_r_ (‘relative LC intensity’, and we also inverted the LC signal values, thus higher values of LC intensity indicate poor LC integrity) for every individual at each time point and observed overall decreases from baseline to follow-up (*t*(76) = −5.69, *P* value < 0.001, 95% confidence interval (CI) −0.04 to −0.02; decreases occur consistently across all ages (Extended Data Fig. 1a,b). Neuropsychological scores were lower at follow-up than baseline, though no significant differences were found in Mini-Mental State Examination (MMSE) (*P* = 0.09, mean difference = 0.26) or Preclinical Alzheimer’s disease Cognitive Composite 5 (PACC5) scores (*P* = 0.12, mean difference = 0.08). At baseline, 74 participants had a Clinical Dementia Rating (CDR) score of 0 and 3 had a CDR score of 0.5. At follow-up, three more participants progressed from CDR 0 to 0.5. At baseline, mean neocortical Aβ = 1.261 distribution volume ratio (DVR) (s.d. = 0.21; Aβ-positivity threshold = 1.324 DVR, Aβ-positive = 10 participants, Aβ-negative = 62 participants; follow-up data from three participants was used for calculating the baseline mean and two participants had missing Pittsburgh Compound B (PiB) values in baseline and follow-up). The ex vivo dataset included 160 MAP participants (Supplementary Table 1b). The MAP sample was divided into two groups: unimpaired participants (*n* = 66, age range 72.72 to 101.19 years, 48 females (72.73%)) and impaired (mild cognitive impairment (MCI) or AD) participants (*n* = 94, age range 74.83 to 99.67 years, 60 females (63.83%)).

### Tau accumulation targets LC before MTL

**Fig. 1:**
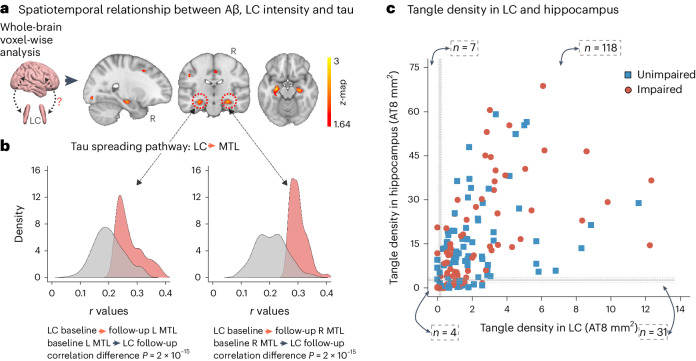
LC integrity predicts tau spreading in subsequent years. **a**, A schematic representation of the neuroimaging analysis between LC integrity (inverted signal) and tau PET images (brain mesh rendered using SurfIce; https://www.nitrc.org/projects/surfice/) (left). Baseline LC integrity (inverted signal) was associated with longitudinal bilateral hippocampus and left amygdala tau (*P* < 0.05 cluster-corrected for multiple comparisons) using whole-brain voxel-wise level GLM analysis (*n* = 77 independent individuals) (right). The brain projection shows one-tailed results (*z*-score > 1.64; the color bar shows the *z*-statistics; cooler colors represent a stronger association). The results are displayed on sagittal, coronal and axial brain views using FSLeyes (FSL, FMRIB). **b**, Each distribution represents the longitudinal relationship between LC integrity (inverted signal) and the tau signal from the voxels within the left or the right MTL clusters surviving the multiple comparisons correction from the previous analysis. These distributions were compared using pairwise *t*-statistics (left cluster, *n* = 186 voxels; right cluster, *n* = 77 voxels). Distributions in red correspond to the tau pathway from baseline LC integrity to follow-up MTL tau; distributions in gray correspond to the pathway from baseline MTL tau to follow-up LC integrity. **c**, Using ex vivo data (*n* = 160 independent individuals), the proportion of low versus high tangle density in LC was tested against the proportion of having low versus high hippocampal tangles (for calculation of the threshold, see Methods; blue dots represent unimpaired participants and red dots represent impaired (MCI and AD) participants). L, left; R, right. Source data

**Extended Data Fig. 2:**
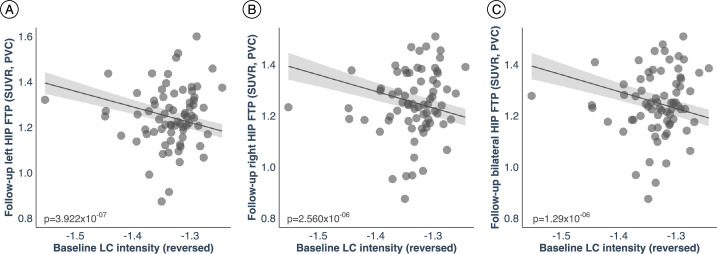
The hypothesized pathological spreading pathway from LC to MTL is supported when adopting a ROI-based approach. **a**) Left hippocampus ROI (n = 77 independent individuals); **b**) Right hippocampus ROI (n = 77 independent individuals); **c**) Bilateral hippocampus (averaged left and right hippocampus ROIs) (n = 77 independent individuals). The scatter plots reflect the association between baseline LC intensity_r_ and tau accumulation in hippocampus at follow-up (robust linear regression, p < 0.05 two-tailed analysis, uncorrected for multiple comparisons). Plots are adjusted for age, sex, CDR and neocortical PiB burden. In all plots, dots represent the individual predicted values of the relationships tested and the shaded areas around the fit lines show 95% CI. Abbreviations: FTP = 18-flortaucipir PET; HIP=hippocampus; p = p value; PVC=partial volume corrected; SUVR=standardized uptake value ratio. Source data

**Extended Data Fig. 3:**
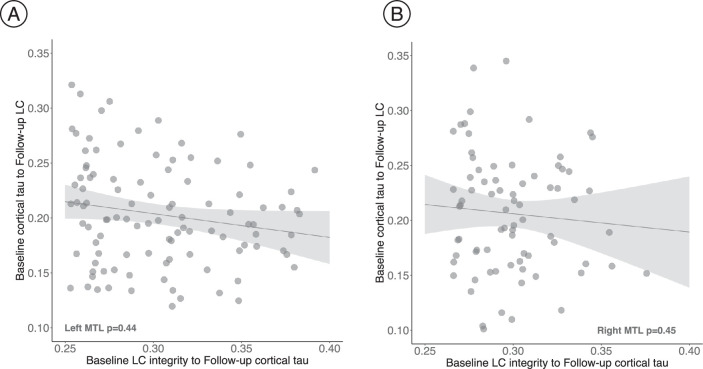
No association was found between our hypothesized tau spreading pathway from LC to MTL, and the reverse model from MTL to LC. **a**) Left MTL (n = 186 voxels). **b**) Right MTL (n = 77 voxels). The scatter plots reflect the association values extracted from the voxels forming the cluster that survived the correction for multiple comparisons when we tested our model (baseline LC integrity to follow-up cortical tau (FTP, SUVR, PVC); robust linear regression, p < 0.05 two-tailed analysis, uncorrected for multiple comparisons). The voxels were kept constant for the reversed model. In all plots, dots represent the estimates (beta-coefficients) of the relationships tested and the shaded areas around the fit lines show 95% CI. Abbreviations: p = p value; LC=locus coeruleus; MTL=medial temporal lobe. Source data

**Extended Data Fig. 4:**
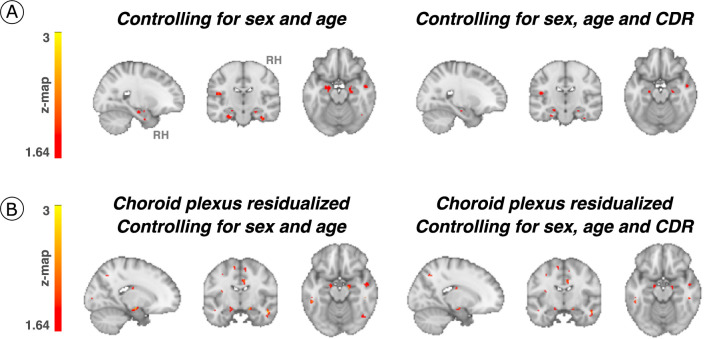
Associations between LC integrity and cortical tau accumulation after controlling for the effect of covariates of no interest. (**a**) Baseline LC integrity (inverted signal) was used as a predictor of longitudinal tau accumulation at the whole-brain voxel-wise GLM level controlling for sex and age or sex, age, CDR (n = 77 independent individuals). The associations between LC integrity and medial temporal lobe regions (for example, hippocampus) were smaller in cluster size but remained significant. (**b**) The choroid plexus FTP-signal was residualized from tau-PET images and added to our associations between LC integrity and cortical tau; then, the additional covariates (sex, age, CDR) were added (whole-brain voxel-wise level GLM analysis) (n = 77 independent individuals). Although the cluster extension was reduced, the associations remained significant. Results are p value < 0.05 cluster-corrected. The brain projection shows one-tailed results (z-score > 1.64; the color bar shows the z-statistics; cooler colors represent a stronger association). In (A) and (B), The results are displayed on sagittal, coronal and axial brain views using FSLeyes (FSL, FMRIB, Oxford, UK). Abbreviations: CDR=Clinical Dementia Rating; RH=Right hemisphere.

First, we tested the hypothesized pathway of pathologic spreading from the LC to brain allo- and neocortical areas using serial LC MRI and tau PET neuroimaging data. We performed voxel-wise regression analyses between inverted LC intensity_r_ (higher indicates worse) and whole-brain tau accumulation flortaucipir (FTP)-PET images. Higher LC intensity_r_ was associated with greater tau deposition in the MTL at baseline and follow-up at the whole-brain voxel-wise level (*P* < 0.05 Monte-Carlo simulation cluster-corrected for multiple comparisons). Thus, worse LC integrity is related to MTL tau accumulation 3 years later (Fig. 1a and Supplementary Table 2). The same association was found between LC intensity_r_ at baseline and region-of-interest-(ROI) left, right or bilateral hippocampus tau accumulation 3 years later (*P* < 0.001; Extended Data Fig. 2). To compare the hypothesized pathway to the reverse pathway (LC intensity_r_ at follow-up and tau deposition images at baseline), we extracted the correlation coefficients of the voxels within the clusters surviving the multiple comparison in both directions and tested distributions differences using *t*-statistics. We found stronger evidence for LC as a predictor of follow-up tau (contrast, ‘MTL tau follow-up ~ LC intensity_r_ at baseline + age + sex’ compared to ‘LC intensity_r_ follow-up ~ MTL tau baseline + age + sex’; distribution difference for left hemisphere cluster *t*(185) = 13.7, *P* < 0.001 95% CI 0.060–0.082; distribution difference for right hemisphere cluster *t*(76) = 13.26, *P* < 0.001 95% CI 0.080–0.108; Fig. 1b). The hypothesized tau spreading pathway (tau spreading from LC to MTL) was not correlated with the reverse model (tau spreading from early affected allocortical regions to LC) (Extended Data Fig. 3). The associations between LC intensity_r_ and voxel-wise tau accumulation remained similar after controlling for sex, age, CDR and neocortical PiB burden, and after residualizing and controlling for the choroid plexus FTP signal (Extended Data Fig. 4).

**Fig. 2:**
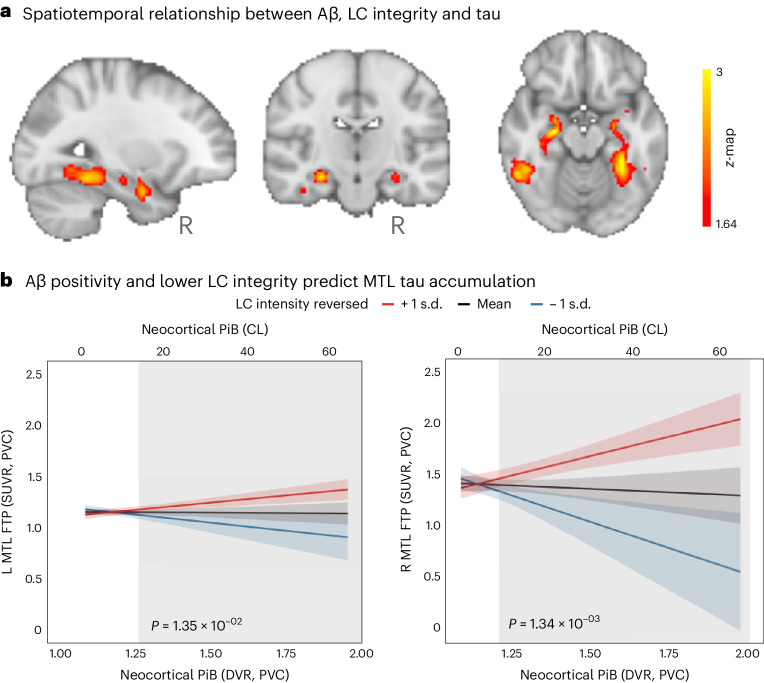
Aβ facilitates LC-related tau spreading beyond MTL regions. **a**, Baseline LC integrity (inverted signal) and Aβ deposition (PiB binding) were synergistically associated with longitudinal bilateral MTL, medial inferior occipito-temporal and posterior occipital tau accumulation (FTP binding) (*P* < 0.05 cluster-corrected for multiple comparisons) using whole-brain voxel-wise level GLM analysis (*n* = 77 independent individuals). The brain projection shows one-tailed results (*z*-score > 1.64; the color bar shows the *z*-statistics; cooler colors represent a stronger association). The results are displayed on sagittal, coronal and axial brain views using FSLeyes (FSL, FMRIB). **b**, Lower LC integrity was related to higher Aβ-related tau accumulation in the MTL approximately 3 years later using robust linear regression analysis (*n* = 75 independent individuals; two-tailed analysis). Then, we used Johnson–Neyman analysis to define the range of PiB values at which the LC–tau association is significant: PiB DVR PVC = 1.26 or 13.94 CL for the left cluster; PiB DVR PVC = 1.20 or 9.67 CL for the right cluster (ranges are indicated by the gray rectangular shadow). Black fit lines represent mean LC integrity, red fit lines indicate +1 × s.d., blue fit lines indicate −1 × s.d. and shaded areas around the fit lines show 95% CI. Source data

**Extended Data Fig. 5:**
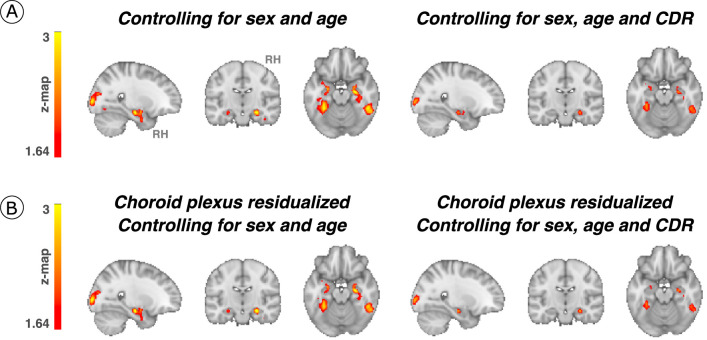
Associations between LC integrity and cortical tau accumulation moderated by neocortical PiB burden after controlling for the effect of covariates of no interest. (**a**) The interaction between baseline LC integrity (inverted signal) and neocortical PiB burden (Aβ) as predictor of longitudinal tau accumulation at the whole-brain voxel-wise level GLM analysis controlling for sex and age or sex, age, and CDR (n = 75 independent individuals). The associations between LC integrity and medial temporal lobe regions (for example, hippocampus) and posterior occipital regions were similar when controlling for age and sex and were smaller in cluster size when controlling for CDR but remained significant. (**b**) The choroid plexus FTP-signal was residualized from tau-PET images and added to our associations between Aβ and LC integrity predicting cortical tau; then, the additional covariates (age, sex, and CDR) were added (whole-brain voxel-wise level GLM analysis) (n = 75 independent individuals). Although the cluster extension was reduced, the associations remained significant. Results are p value < 0.05 cluster-corrected). The brain projection shows one-tailed results (z-score > 1.64; the color bar shows the z-statistics; cooler colors represent a stronger association). In (A) and (B), the results are displayed on sagittal, coronal and axial brain views using FSLeyes (FSL, FMRIB, Oxford, UK). Abbreviations: CDR=Clinical Dementia Rating; RH=Right hemisphere.

Given that Aβ is known to facilitate tau spreading^9^, we examined the moderating effect of Aβ and observed that at a given level of Aβ deposition, higher LC intensity_r_ at baseline was associated with greater tau accumulation at follow-up in bilateral MTL (hippocampus and amygdala), and regions beyond the MTL, such as the medial inferior occipito-temporal (parahippocampus and fusiform cortex) and posterior occipital cortices (*P* < 0.05 Monte-Carlo simulation cluster-corrected for multiple comparisons) (Fig. 2a and Supplementary Table 3). Johnson–Neyman analysis revealed that these positive associations between baseline LC intensity_r_ and tau deposition at follow-up (Fig. 2a) emerged at Aβ levels below the established Aβ-positivity threshold of 1.324 DVR, partial volume correction (PVC) or 18.49 centiloid (CL) (left MTL, Aβ ≥ 1.26 (PiB DVR and PVC) or 13.94 CL and right MTL, Aβ ≥ 1.20 (PiB DVR and PVC) or 9.67 CL) (*P* < 0.05) (Fig. 2b). These relationships did not change after controlling for sex, age, CDR and after residualizing and controlling for choroid plexus FTP signal (Extended Data Fig. 5).

**Extended Data Fig. 6:**
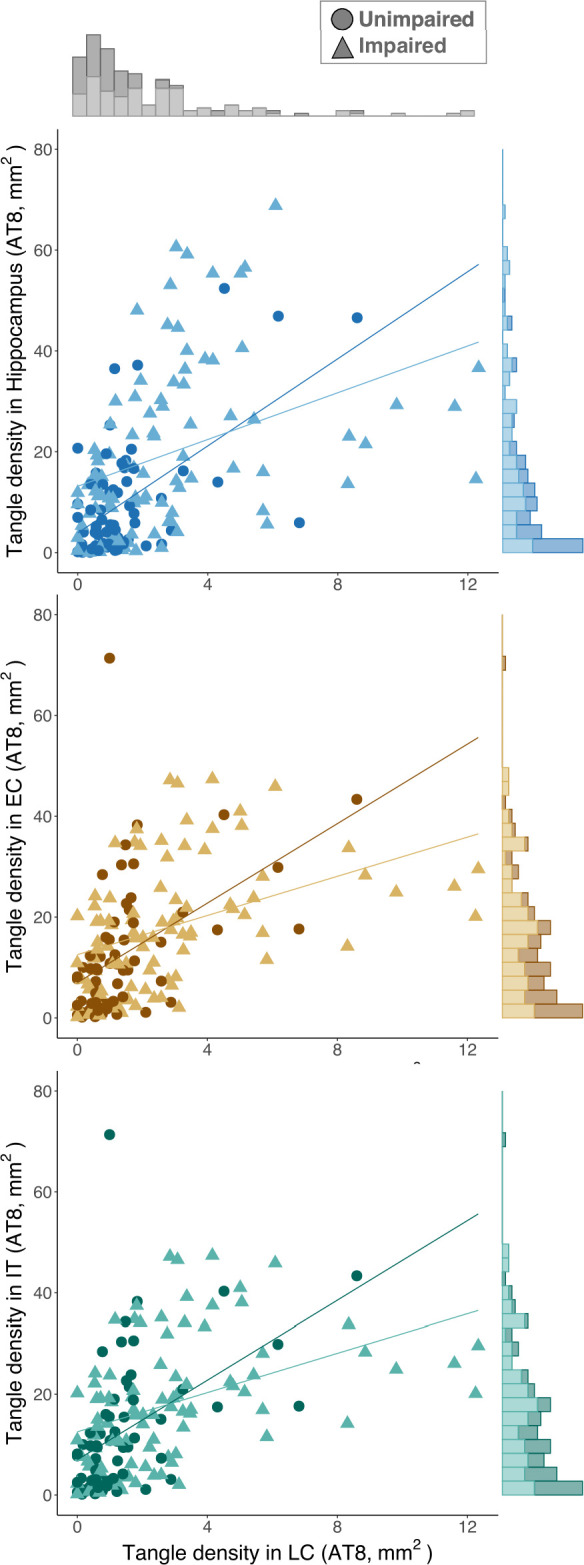
LC tangle density is related to tangles in MTL areas. In unimpaired participants (EC, N = 65; Hippocampus, N = 66; IT, N = 66; LC, N = 66 independent individuals represented by dots), LC tangle density is related to tangles in MTL structures, such as the hippocampus (upper plot) and the entorhinal cortex (middle plot), and the inferior temporal cortex (lower plot). These associations are consistent with the pattern observed in impaired (EC, N = 93; Hippocampus, N = 94; IT, N = 94; LC, N = 94 independent individuals with mild cognitive impairment or AD; represented by triangles) participants. Lines in the scatter plots represent linear trends. LC tangle density histogram appears on the upper part of the plots, while hippocampus, entorhinal cortex, or inferior temporal cortex tangle density histograms appear on the right-side of the respective plots.

**Extended Data Fig. 7:**
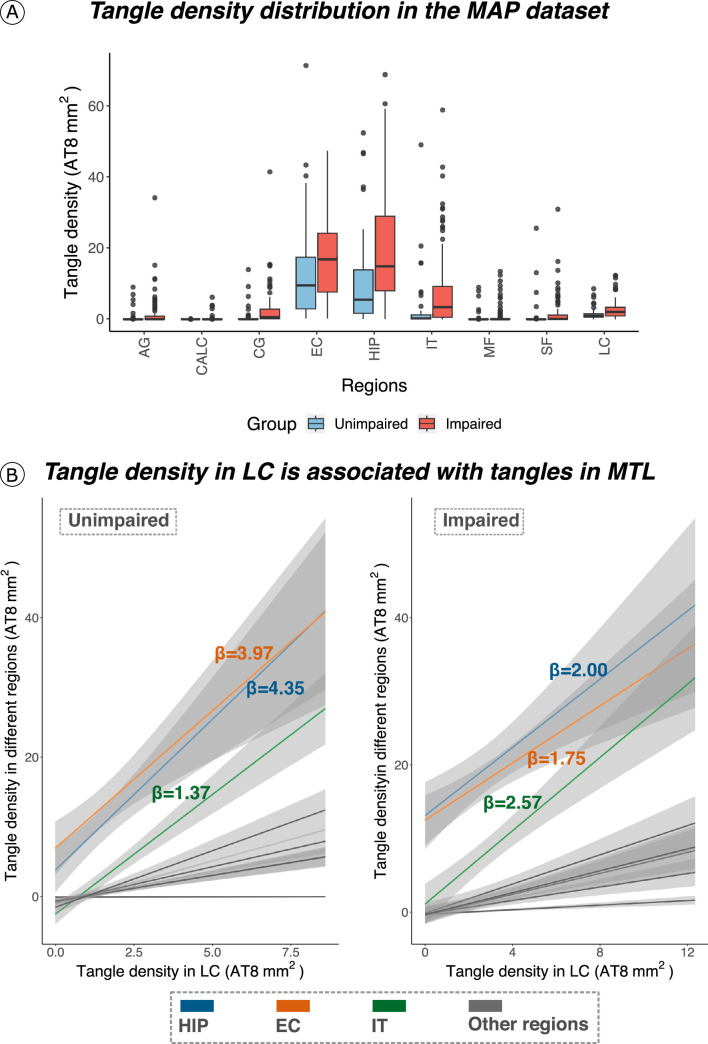
Tangle density in LC is associated with early affected tau cortical regions. (**a**) Tangle density distribution in the MAP dataset. In both unimpaired and impaired (MCI/AD) individuals, tangle density is higher in the MTL (EC and hippocampus) compared to other regions (the boxplots covers the interquartile range (IQR): the centerline of the boxplot corresponds to the median (Q2), the lower and upper bound of the boxplots correspond to the 25th percentile (Q1) and the 75th percentile (Q3) respectively, the error bar minimum value is the minimum value in the data (Q1–1.5*IQR) and the maximum value is the maximum value in the data (Q3 + 1.5*IQR). (**b**) Tangle density in LC is strongly related to tangle density in MTL (that is, hippocampus and entorhinal cortex), and in IT (restricted robust linear regression with age, sex and postmortem interval as covariates, two-tailed analysis, all p < 0.05; regression lines represent slope between the relationships tested and the shaded areas around the fit lines show 95% CI). Sample size for (A) and (B) (unimpaired | impaired): AG = 65|94 individuals; CALC = 66|94 individuals; CG = 66|94 individuals; EC = 65|93 individuals; HIP = 66|94 individuals; IT = 66|94 individuals; MF = 66|94 individuals; SF = 66|94 individuals; LC = 66|94 individuals. Abbreviations: AG=angular gyrus; CALC= calcarine; CG=cingulate cortex; EC=entorhinal cortex; HIP=hippocampus; IT=inferior temporal cortex; LC=locus coeruleus; MF=midfrontal gyrus; SF=superior frontal gyrus.

Spearman rank partial correlation analyses (Extended Data Fig. 6) in the MAP dataset showed that in unimpaired participants, LC tangle density was related to tangles in MTL structures: hippocampus (rho = 0.51, *P* < 0.001, *n* = 62) and entorhinal cortex (EC) (rho = 0.57, *P* < 0.001, *n* = 61). Also, LC tangle density was related to tangle density in the inferior temporal (IT) cortex (rho = 0.47, *P* = 0.0003, *n* = 59). Similar results were observed in impaired individuals (hippocampus (rho = 0.48, *P* < 0.001, *n* = 90), EC (rho = 0.52, *P* < 0.001, *n* = 89) and IT cortex (rho = 0.68, *P* < 0.001, *n* = 89). Correlations were adjusted for LC neuronal density, sex, age, postmortem interval and, in a second step, for global Aβ burden (Supplementary Table 4). See Extended Data Fig. 7 for associations between LC tangle density and tangle density in other regions.

Based on the in vivo imaging results, we approximated a spatial staging of LC tangles versus hippocampal tangles in the MAP data (Fig. 1c). The maximum density of hippocampal tangles of all individuals in Braak stage II or lower was identified (cutoff of 7.91); data from unimpaired participants below this value (*n* = 39) was used to calculate the mean of 2.757 (95% CI 2.027–3.486; Methods). The binomial exact test results indicated that the probability of having elevated tau tangles in LC but not the hippocampus is 20% (95% CI 0.14–0.27), the likelihood of having high tau tangles in the hippocampus but not in LC, 0.05% (95% CI 0.02–0.09) and in both regions is 76% (95% CI 0.68–0.82); supporting the hypothesis of tau spreading from LC to the allocortical areas. Individuals with elevated tangle density in the LC and in Braak stage III regions also had a significantly greater Aβ burden than individuals with elevated tau tangles only in LC (*t* = 3.78, *P* < 0.001, 95% CI 1.25–4.02) or individuals with low tangle densities in both the LC and Braak stage III regions (*t* = 5.77, *P* < 0.001, 95% CI 2.34–5.50).

### The LC–MTL tau pathway is associated with cognitive decline

**Fig. 3:**
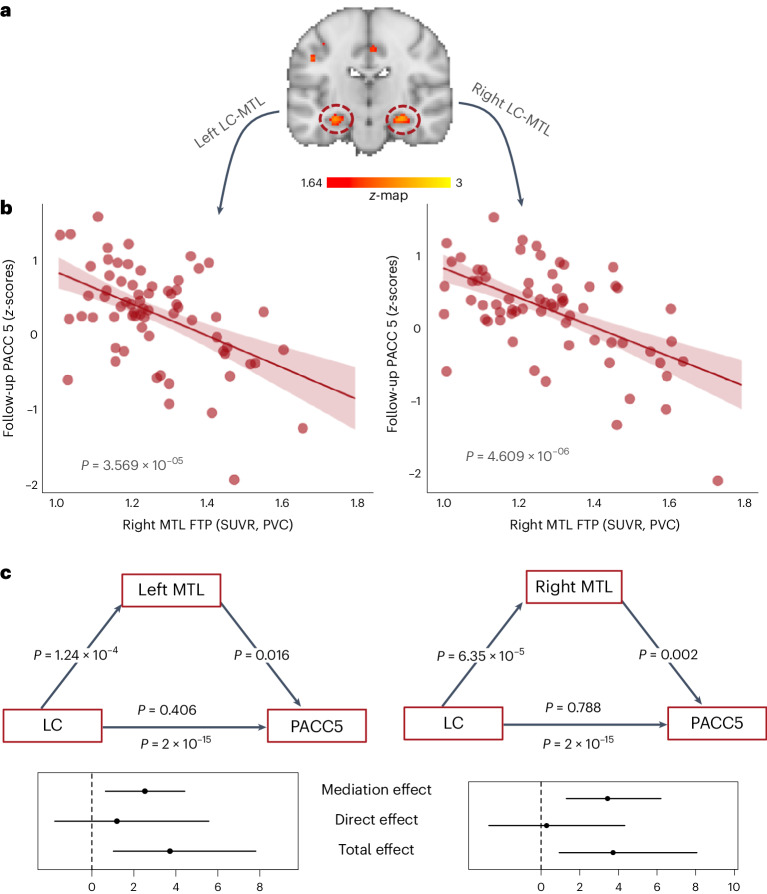
Cognitive outcomes are predicted by the biological link between LC and MTL tau burden. **a**, The individuals’ LC-related follow-up MTL tau values were used as predictors of cognitive performance (averaged voxel FTP values (SUVR and PVC) extracted from the clusters surviving multiple comparison correction after conducting whole-brain voxel-wise level GLM analysis (*n* = 77 independent individuals). The brain projection shows one-tailed results of the whole-brain (*z*-score > 1.64; *P* < 0.05, cluster-corrected for multiple comparisons; the color bar shows the *z*-statistics, where cooler colors represent a stronger association). The results are displayed on a coronal brain view using FSLeyes (FSL, FMRIB). **b**, Higher LC-related MTL tau accumulation was associated with lower cognitive performance as measured by the PACC5 approximately 3 years later (*n* = 74 independent individuals). The plots reflect the relationship between tau accumulation and PACC5 *z*-scores adjusted by age, sex, years of education and CDR (robust linear regression and two-tailed analysis). Dots represent the individual predicted values of the relationship tested and the shaded areas around the fit lines show 95% CI. **c**, Follow-up MTL tau (FTP, SUVR and PVC) mediated the relationship between baseline LC intensity_r_ and follow-up PACC5 performance (*z*-scores) 3 years later (two-tailed mediation analysis). The graphical representation of the mediated relationship displays the *p*-values of the estimates, while the forest plots depict the *β*-coefficients along with the 95% CI (*n* = 74 independent individuals). Source data

**Extended Data Fig. 8:**
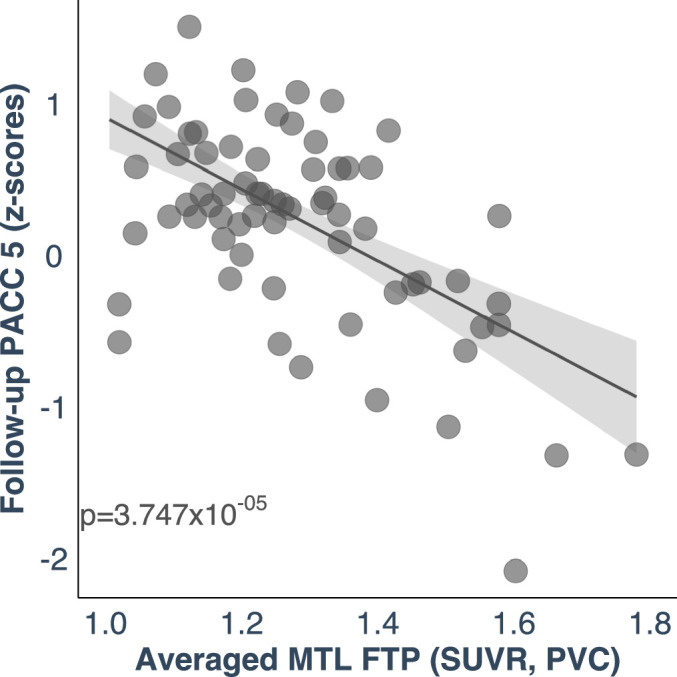
Cognitive outcomes are predicted by the biological link between LC and MTL tau burden. A higher correlation between LC-related - MTL tau accumulation was associated with lower cognitive performance as measured by the PACC 5 (z-scores) approximately three years later (n = 77 independent individuals). The plot reflects the relationship between the averaged left and right MTL tau deposition and PACC5 z-scores adjusted by age, sex, years of education and CDR (robust linear regression, two-tailed analysis). Dots represent the estimates (beta-coefficients) of the relationships tested and the shaded areas around the fit lines show 95% CI Abbreviations: FTP = 18-flortaucipir PET; LC=locus coeruleus; MTL=medial temporal lobe; PACC5=Preclinical Alzheimer’s disease Cognitive Composite 5; p = p value; PVC=partial volume corrected; SUVR=standardized uptake value ratio. Source data

**Extended Data Fig. 9:**
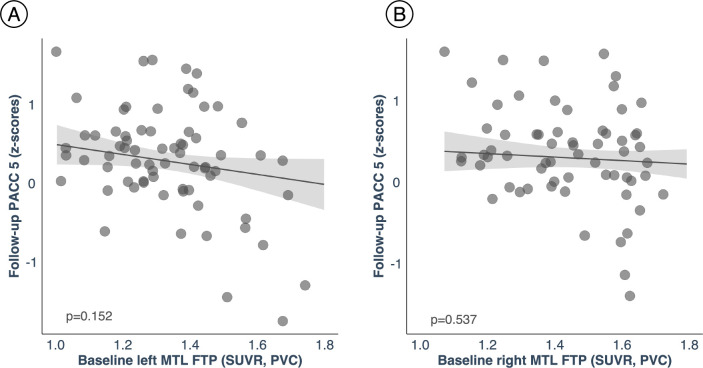
Cognitive outcomes are not associated with the reverse hypothesized pathway ‘follow-up LC-related – baseline MTL tau deposition’. (**A**) Left MTL (n = 74 independent individuals). (**B**) Right MTL (n = 74 independent individuals). The possible association between tau deposition in the MTL at baseline with LC integrity (inverted signal) years later does not predict lower cognitive performance on the PACC5 (z-scores). The plots are adjusted by age, sex, years of education and CDR (robust linear regression, two-tailed analysis). In all plots, dots represent the estimates (beta-coefficients) of the relationships tested and the shaded areas around the fit lines show 95% CI. Abbreviations: FTP = 18-flortaucipir PET; MTL=medial temporal lobe; p = p value; PACC5=Preclinical Alzheimer’s Cognitive Composite 5; PVC=partial volume correction; SUVR=standardized uptake value ratio. Source data

Using robust linear regression analysis, we observed that our hypothesized tau spreading pathway is associated with worse cognitive performance approximately 3 years later. Specifically, using the LC-related MTL tau values (Fig. 3a), we observed that greater LC-related MTL tau burden leads to worse PACC5 performance (Fig. 3b and Supplementary Tables 5 and 6). The results were similar when averaging the left and right MTL FTP values (Extended Data Fig. 8). To model the relationships between the hypothesized pathway and cognitive performance, we used path analyses and found that follow-up MTL tau mediates the relationship between baseline LC intensity_r_ and follow-up PACC5 performance (5,000 simulations; left MTL: mediation effect, *β* = 2.78, *P* = 0.01, 95% CI 0.46–5.36; total effect, *β* = 4.88, *P* < 0.001, 95% CI 2.05–9.32; and proportion mediated, *β* = 0.57, *P* = 0.01, 95% CI 0.09–1.38; right MTL: mediation effect, *β* = 3.67, *P* < 0.001, 95% CI 1.14–6.75; total effect, *β* = 4.88, *P* < 0.001, 95% CI 2.10–9.39; and proportion mediated, *β* = 0.75, *P* < 0.001, 95% CI 0.24–1.68; Fig. 3c). For the reverse model, MTL tau at baseline did not predict follow-up PACC5 performance (Extended Data Fig. 9).

### A shared genetic background between LC and limbic areas

**Fig. 4:**
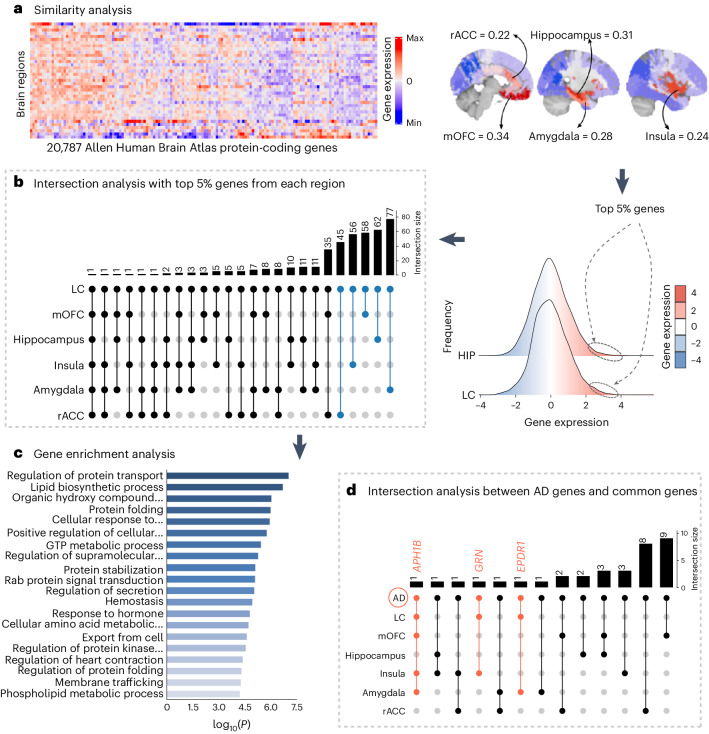
Neurogenetic approach exploring common genetic background across the brain. **a**, A whole-brain region-wise phenotypic-transcriptomic similarity analysis using the AHBA was conducted correlating LC to 68 regions from the Desikan–Killiany neocortical parcellation, the hippocampus and the amygdala. The sagittal brain slices (FSLeyes; FSL, FMRIB) show LC’s gene expression profile similarity to that of the hippocampus, amygdala, insula, mOFC and rACC (warmer colors indicate higher similarity). The genes with the highest genetic expression (top 5%) within these six regions were selected for subsequent analysis (the distribution corresponding to the genetic expression of protein-coding genes colocated at the LC and the hippocampus are shown). **b**, An intersection analysis was used to define common protein-coding genes between LC and each of the other regions, aggregating genes involving LC plus one region (*n* = 298 protein-coding genes). **c**, GO enrichment analysis revealed that among the biological functions related to these genes, which are highly expressed in early AD-affected regions, regulation of protein transport was found to be the main term (thresholds for terms, *P* < 0.01, count > 3 and enrichment factor > 1.5; *P* values, log_10_(*P*) are calculated based on the cumulative hypergeometric). **d**, An intersection analysis was used to find, within the common gene expression profiles (*n* = 298 protein-coding genes), genes related to AD (*n* = 75 genes). The *APH1B*, *GRN* and *EPDR1* genes were found within the AD-related genes and our neurogenetic approach results. GTP, guanosine triphosphate; HIPP, hippocampus.

**Extended Data Fig. 10:**
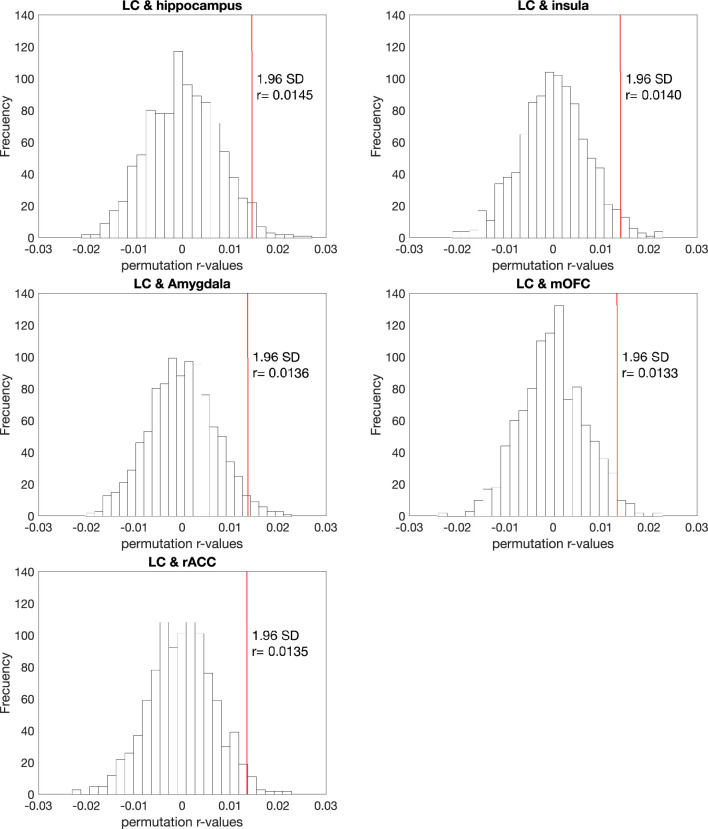
The relationship between the genetic profile of the locus coeruleus and the top 5 most correlated brain regions remained robust after permutation analysis. The vertical solid red line represents the r-value corresponding to 1.96 standard deviation. Abbreviations: LC=locus coeruleus; mOFC=medial orbitofrontal cortex; r = r-value; rACC=rostral anterior cingulate cortex; SD=standard deviation.

Using the AHBA and Gene Ontology (GO) functionality resources, we investigated whether the LC has a similar gene expression profile to other areas in the human brain (Fig. 4a, left) to shed light on the specific biological characteristics of LC-related tau accumulation in the human brain. We observed highly similar protein-coding gene expression levels between the LC and limbic system structures, including the hippocampus (*r* = 0.31; *P* < 0.001), the amygdala (*r* = 0.24; *P* < 0.001), the rostral anterior cingulate cortex (rACC; *r* = 0.22; *P* < 0.001), the medial orbitofrontal cortex (mOFC; *r* = 0.34; *P* < 0.001) and the insula (*r* = 0.28; *P* < 0.001) (Fig. 4a, middle). The significance of these associations remained robust after the permutation analysis (Extended Data Fig. 10). Next, the top 5% of the protein-coding genes colocated between the LC and each of these regions (Fig. 4a, right) were used in subsequent intersection analysis and resulted in 298 identified common genes across areas (LC ∩ hippocampus = 62 genes; LC ∩ amygdala = 77 genes; LC ∩ rACC = 35 genes; LC ∩ mOFC = 58 genes; and LC ∩ insula = 56 genes) (Fig. 4b and Supplementary Table 7). This final list (*n* = 298 genes) was introduced in Metascape as a *Homo* *sapiens* list of genes. The enrichment exploration of the GO functionalities behind the gene list showed a common biological background devoted to one main term: regulating protein transport (Fig. 4c and Supplementary Table 8). Concerning the AD-related genes, the analysis yielded three genes: *APH1B* gene common to AD ∩ LC ∩ mOFC ∩ insula ∩ amygdala; *GRN* gene common to AD ∩ LC ∩ insula; *EPDR1* gene common to AD ∩ LC ∩ amygdala (Fig. 4d). The random permutation analysis determined that the probability of identifying three AD-risk genes within the LC–MTL–limbic common genes was 3% (Supplementary Table 9).

## Discussion

Identifying the earliest pathways of tau propagation in AD-related pathology is critical for interventions aiming to halt the spreading of tau and the associated onset of clinical symptoms. In the current study, we examined whether the LC is one of the first regions of tau accumulation and spreading to the cortex and what factors contribute to its specific vulnerability to early AD-related changes. Consistent with autopsy observations, we demonstrated that reduced LC integrity precedes tau accumulation in the MTL and that this hypothesized pathway of tau spreading was related to lower cognitive performance approximately 3 years later. Notably, while LC integrity-related associations were restricted to tau accumulation in the MTL in our main analyses, we observed that as Aβ increases, LC-related tau accumulation patterns involve regions outside the MTL, stretching into inferior occipito-temporal and posterior brain regions, which is consistent with current disease models^3,15^. While tau spreading is widely investigated, the mechanisms underlying propagation of pathology remain not well known. Our genetic-imaging intersection analyses set a first step in identifying transcriptomic profiles of protein transport regulation and protein folding as factors contributing to tau vulnerability for this specific pathway. These findings support the LC as a spatiotemporal epicenter for spreading tau pathology from local subcortical areas to disseminated allo- and neocortical systems and contributing to the increased risk of AD.

Several autopsy studies reported evidence of abnormal pre-tangle material in the LC before any allo- and neocortical involvement of either tau or Aβ, and before the emergence of clinical symptomatology^3,5,6,16–19^. But what remained unclear and debated is whether the LC is one of the initial locations from where tau spreads to allo- and neocortical regions or whether seeding occurs first in the EC^8^. Animal studies provided evidence for tau spreading from the LC to other regions. Rat models expressing hyperphosphorylated tau in the LC exhibited spreading of tau from the LC to the raphe nucleus at 4 months, followed by learning difficulties and reduced axonal density at 7 months^20^. Observations at later time points in these rats, or lesioning the LC in transgenic mice models, revealed exacerbated impairment on memory tasks, LC neuronal loss and hippocampal neurodegeneration^20–22^.

Examining tau spreading in vivo in humans is unfortunately hampered by the off-target binding of current PET radioligands to neuromelanin and the limited spatial resolution of PET cameras. Fortunately, our previous work indicated that LC integrity obtained from dedicated MRIs could signal tau-related processes^9^. Taking advantage of longitudinal MRI and PET imaging, our results now support observations from autopsy and animal studies. They show that neurodegenerative and tau-related processes in the LC impact future tau accumulation in MTL regions, and when Aβ is elevated, LC-related tau accumulation progresses to lateral tempo-occipital regions, following the topographical progression described in Braak staging. This spreading outside the MTL starts at subthreshold Aβ values (10–14 CL), indicating that this LC–MTL pathway of pathologic changes occurs early in the disease cascade and that spreading to neocortical regions is further facilitated by increasing levels of Aβ. The observations in the MAP data confirm this spatiotemporal pattern, as the likelihood of following the Braak staging was greater than being in discordant stages, and Aβ burden is higher when tau accumulation has reached at least Braak stage III. Notably, these patterns remained similar when controlling for LC neuronal density, corroborating evidence that even though LC volume loss occurs early, neuronal loss in the LC is more likely to occur from the prodromal AD stage^23^. The preferential topography of initial tau spreading from the LC to the hippocampus and amygdala is consistent with the topographical organization of the rostral LC’s dense efferent projections to the MTL and its functional specificity^24^.

We and others have demonstrated previously that poor LC integrity predicts worse memory performance and AD-related memory decline^9,25–28^. The involvement of the MTL in these associations is critical, as it signals a transition to preclinical AD, a time when cognitive symptoms become evident^1,29^. Long-term potentiation, the neurobiological process underlying memory and learning, also depends on noradrenergic modulation^30^. Indeed, the LC–NE system largely modulates all stages of memory (learning, consolidation and retrieval) as NE is released in different structures of the MTL and forebrain^31^. Animal studies demonstrated that accumulation of hyperphosphorylated tau in the LC was associated with impaired hippocampal-mediated memory, reduced hippocampal NE levels and decreased LC fiber density in the MTL^20,21,32^. Following these observations, we speculate that reduced integrity of the LC would result in a shortage of NE in several brain structures, compromising learning and cognitive functioning. The perturbation of the LC–NE pathway to the MTL (due to tau pathology) would lead to dysregulation of neurotransmitter signaling, subsequently causing impaired functioning of the memory system. Similarly, TgF344 rats, which display age-related endogenous tau pathology in the LC at 6 months, exhibited progression of tau pathology to the entorhinal and hippocampus 10 months later, along with reduced NE levels in the hippocampus. At 16 months of age, these rats also exhibited impaired spatial reversal learning, but notably designer receptors exclusively activated by designer drugs (DREADD)-stimulation of the LC rescued learning in these animals^32^. Specific activation patterns of the LC were associated with the maintenance of memory performance, as well as LC fiber density^33^, suggesting that targeting the signaling capabilities of the LC in the earliest stages of the disease may be critical. Our results align with this and demonstrate the urgency of maintaining LC health and halting the spreading of tau to the MTL to delay cognitive decline.

The coexpression of gene patterns between the LC and hippocampus, amygdala, insula, mOFC and rACC revealed several biologically meaningful profiles that could underlie the vulnerability to the hypothesized LC–MTL tau spreading pathway. We detected three recently identified AD-risk genes (*APH1B*, *GRN* and *EPDR1*)^34,35^. Furthermore, GO enrichment analysis classified several of the 298 coexpressed genes as those involved in regulating protein transport. Three of these genes may play a role in tau accumulation and spreading. For example, misfunction of the *BAG3* gene leads to increased pathological tau accumulation in excitatory cells, whereas its overexpression reduces tau accumulation in inhibitory cells^36,37^. The second is the *MAP1B* gene, in which Aβ binding to peptides that comprise the microtubules has been associated with impairment of microtubule-dependent transport, loss of neuronal cytoskeletal integrity and synaptic dysfunction^38^. Notably, the *CDC42* gene regulates glycogen synthase kinase 3 (GSK3) proteins encoded by *GSK3α* and *GSK3β* genes. The *GSK3β* gene is a fundamental regulator of cellular processes such as microtubule–cytoskeleton reorganization, neuronal polarity and neuronal migration by phosphorylating proteins like *MAP1B* and tau, among other microtubule-associated proteins^39–43^. Dysregulation of *GSK3β* activity in neurons has been linked to the pathology observed in different neurodegenerative diseases^39,40^. Notably for early AD pathogenesis, it has been found that oligomers of Aβ can take over and rewire NE, signaling leading to the activation of the pathogenic *GSK3β*–tau cascade^44^. In addition, downregulation of the *MAP1B* gene in the LC has been observed in MCI and AD and was associated with worse cognitive performance, indicating that LC neurons undergo axonal neurodegeneration during the prodromal stages^45^. This is consistent with the previously discussed animal studies reporting reduced LC fiber density due to tau accumulation^20,21,32^. The following two gene functionalities found in this study, were ‘lipid biosynthetic processes’ and ‘protein folding,’ which can be related to tau and Aβ propagation across the neural system, including LC^46–50^. Lipid metabolism has been previously related to AD pathophysiology, specifically to tau and Aβ propagation across the neural system^47,48^. Apolipoprotein E (*ApoE* gene) is a lipid-related protein-coding gene with a fundamental role in the catabolism of lipidic lipoprotein constituents. In recent animal studies, ApoE ε4 has been linked to tau pathology, after studying its binding relation to vesicular monoamine transporters (such as *SLC18A2* or *VMAT2* genes) and its inhibiting action to vesicular NE uptake. This in turn leads to increased 3,4-dihydroxyphenyl-glycolaldehyde (DOPEGAL) produced exclusively by monoamine oxidase A (*MAO-A* gene) in noradrenergic neurons^51^. DOPEGAL activates asparagine endopeptidase (AEP), which facilitates the aggregation and propagation of tau and cleaves amyloid precursor protein (*APP* gene) and microtubule-associated protein tau (*MAPT* gene). Thus, elevated DOPEGAL exacerbates LC degeneration and tau spreading^46,52^. Finally, concerning the functionality protein folding in the context of AD, recent work reported that NE can disrupt tau filaments leading to tau degradation^46,49,50^.

This study has several limitations. The biological interpretation of the MRI-based LC integrity measure is still under investigation. Given the increasing amount of data showing a greater rostro-dorsal vulnerability in the LC^51^, we believe that future studies examining the full length of the LC using 7T MRI will be essential to understand possible heterogeneity in the LC regarding its involvement in initial spreading of AD pathology. Our previous work demonstrated a strong correlation between LC integrity and tau deposition in both autopsy and in vivo data^9^, suggesting a contribution of tau-related processes. The current findings are consistent with this, though neuromelanin, water, lipids^53,54^ and other macromolecular elements are likely also playing a role. Neuropathology–imaging correlations are needed to disentangle the contribution of tau and other potential biological sources to the MRI signal. Second, our sample size was modest and included individuals in the early stages of the disease, limiting our evaluation of tau spread beyond Braak stage III. Future studies should include individuals with greater cognitive impairment and pathology variability to understand whether tau spread in later stages is independent of, synergistic with or solely driven by Aβ. Future studies with a longer follow-up time will also make it possible to examine nonlinear associations and incorporate growth curve models to model within-person patterns of change. It is essential to recognize that although longitudinal data do not provide conclusive causal information, it contributes critical knowledge in our endeavor to understand the temporal chain of pathologic events in AD. In relation to tau measurements, we acknowledge that in vivo tau radioligands detect tau fibrillar forms; therefore, we cannot exclude any potential early seeding from soluble forms of tau from other regions. Furthermore, the use of the AT8 antibody to measure tau deposits in postmortem brains limits the count of tau tangles to intracellular tangles, missing the extracellular tangles from the count. We acknowledge the exploratory nature of our neuroimaging-genetic approach, which was conducted using expression values from cognitively unimpaired individuals. Therefore, the outcomes of our analysis should be interpreted cautiously and replication using brain transcriptomic information covering the whole AD spectrum, ideally with direct correspondence with in vivo or ex vivo imaging, is warranted to conclusively make inferences on the specific biological features underlying the vulnerability of early tau pathways from the LC to the cortex.

To conclude, using a multimodal and multilayered approach with in vivo LC and tau neuroimaging data, we found that lower LC integrity preceded and was associated with the spreading of tau to MTL structures and conjointly predicted lower cognitive performance. The early vulnerability of the LC and the topography with MTL tau motivated the search for a shared anatomic genetic background relevant to AD pathophysiology. Our analyses revealed a selective vulnerability of genetic coexpression profiles between LC, MTL and limbic regions of processes mapping to tau formation, possible tau spreading and associated axonal stability pathways. These findings provide critical insight into the spatiotemporal patterns and molecular basis of initial tau spreading involving the LC and emphasize the importance of early interventions mitigating tau spread.

## Methods

### Participants

#### In vivo dataset

A total of 77 middle-aged-to-older individuals from the Harvard Aging Brain Study (HABS) and the affiliated Locust study with longitudinal follow-up were included in this study (Supplementary Table 1a). All participants underwent 3T MRI imaging, including our dedicated LC sequence^9^ and amyloid and tau PET imaging. Inclusion criteria required a CDR global score of 0 at baseline, a MMSE score equal to or above 25 and performing the Logical Memory Score Delayed-Recall Test within education-adjusted norms (>10 for ≥16 years of education, >6 for 8–15 years of education and >4 for <8 years of education). As tau PET and LC imaging protocols were introduced recently into the HABS study, three participants had progressed to CDR = 0.5 at baseline LC imaging and three more progressed at follow-up. The presence of clinical depression (geriatric depression scale) below 11 of 20 (ref. ^55^) or other psychiatric illnesses, history of alcoholism, drug abuse or head trauma were considered exclusion criteria. The Partners Human Research Committee approved the research protocols of Massachusetts General Hospital (Institutional Review Board agreement nos. 2019P001137, 2010P000297 and 2020P001930). All participants provided written informed consent and received monetary compensation after each visit.

#### Rush MAP dataset

We investigated 160 participants from the MAP^13,14^ (Supplementary Table 1b), an ongoing longitudinal clinicopathological study that started in 1997. The eligibility criteria were age 55 years or older, absence of a previous dementia diagnosis and consent to annual clinical evaluation and brain autopsy at death. Participants were recruited from retirement communities, social service agencies, subsidized housing facilities and individual homes in the Chicago metropolitan region. This sample included individuals for whom detailed LC neuropathology data were available and consisted of 66 individuals with normal cognition and 94 individuals with MCI or AD at their last clinical visit before autopsy^56^. The initial diagnosis was made each year by a neuropsychologist and clinician and the final diagnosis was established by a neurologist blinded to postmortem data based on the National Institute of Neurological and Communicative Disorders and Stroke and the Alzheimer’s Disease and Related Disorders Association criteria^56–58^. All data were shared with a Data User Agreement. An institutional review board of Rush University Medical Center approved the study. All participants signed an informed consent, an Anatomical Gift Act and a repository consent that allowed their data to be shared.

#### AHBA dataset

The AHBA^59,60^ is a transcriptional atlas of the adult human brain derived from histological analysis and microarray profiling. Data were originally obtained from six donors between 18 and 68 years of age, with no known neuropsychiatric or neuropathological history^60^. The transcriptome dataset consists of genetic expression of 20,737 protein-coding genes extracted from 58,692 measurements from 3,702 brain samples. The samples were initially mapped to native three-dimensional MRI coordinates, then to the Montreal Neurological Institute (MNI) coordinate space. Each donor’s closest blood relative provided informed consent for brain tissue collection.

### Imaging data acquisition and preprocessing

#### Structural MRI acquisition and preprocessing

MRI studies were performed at the Massachusetts General Hospital, Athinoula A. Martinos Center for Biomedical Imaging, on a 3T imaging system (TRIM Trio, Siemens). Participants were reinforced to stay still and a short acquisition time was used to minimize motion (for more details, see previous work^9^). The MRI protocol included a structural 3D T1-weighted volumetric magnetization–prepared rapid acquisition gradient-echo images (repetition time = 2,300 ms, echo time = 2.95 ms, inversion time = 900 ms, flip angle = 9° and 1.05 × 1.05 × 1.20-mm resolution) and an optimized MRI acquisition for locating the LC (a two-dimensional T1-weighted turbo-spin-echo sequence with additional magnetization transfer contrast; repetition time = 743 ms, echo time = 16 ms, flip angle = 180°, six slices, four online averages, 0.4 × 0.4 × 3.00-mm resolution and an acquisition time of 3 min and 22 s). Areas of interest (LC and reference region and pontine tegmentum) were registered to each individual using a combination of high-dimensional diffeomorphic with rigid-body registrations. Each slice containing the LC area was normalized to the pontine tegmentum. LC signal intensity (an indicator of LC integrity) was quantified as the mean intensity from five contiguous voxels with the highest values within LC ROIs following 30 search iterations. To facilitate the interpretation of LC signal intensity relative to the PET biomarker data, we inverted the signal values (higher values of LC intensity indicate poor LC integrity) and we refer to it as ‘LC intensity_r_.’ Postmortem studies have not reported asymmetry in LC tau deposition or neuronal changes in LC and to keep consistency with other studies, the left and right signals were averaged^9^.

For all T1 images, the automated reconstruction protocol of FreeSurfer (v.6.0.0) was performed as described previously^9,61^. This protocol includes (1) automated segmentation; (2) intensity normalization; (3) skull stripping; (4) separating left and right hemispheres; (5) excluding brainstem and cerebellum; (6) correcting topology defects; (7) defining the borders between gray matter, white matter and cerebrospinal fluid; (8) parcellating allo- and neocortical and subcortical areas; and (9) visually inspecting images and, if necessary, editing them.

#### Molecular PET image acquisition and preprocessing

We used [^11^C]PiB PET and [^18^F]FTP PET) imaging acquired at Massachusetts General Hospital, on a Siemens/CTI ECAT HR+ scanner. PiB PET was acquired with a bolus injection (8.5–15 mCi), followed immediately by a 60-min dynamic acquisition in 69 frames (12 × 15 s and 57 × 60 s). FTP PET was acquired from 75–105 min after bolus injection (9.0–11.0 mCi) in 4 × 5-min frames.

#### PiB PET preprocessing

PiB PET retention intensity was expressed as the DVR with cerebellar gray matter as a reference tissue using the Logan graphical method applied to data over the 40–60-min post-injection integration intervals^62^. Neocortical PiB retention was assessed in a large neocortical ROI aggregate, including frontal, lateral temporal and retrosplenial cortices (FLR). Aβ status was ascertained in this FLR region using a previously determined cutoff value based on the Gaussian mixture modeling approach cutoff value of 1.324 DVR^9^. Regional PiB PET data underwent PVC using the geometrical transfer matrix (GTM) method as implemented in FreeSurfer^63^, assuming an isotropic 6-mm point spread function.

#### FTP PET preprocessing

FTP PET data were reconstructed by applying standard data corrections^64^. Each frame was evaluated to verify adequate count statistics and motion correction was applied using an automated frame-to-frame realignment algorithm and visually checked. To assess the anatomy of allo- and neocortical FTP binding, each individual PET dataset was rigidly co-registered to the individual’s MPRAGE data. Structural images were normalized to the MNI space. Using the MNI atlas, the cerebellar gray matter was used as the reference region and a mask covering the entire cerebral cortex and subcortical gray matter regions was used in our neuroimaging analyses (MNI152, 2 mm^3^ isotropic). Consistent with our previous work^47,65^, we used the cerebellar gray matter as the reference region as it seems less confounded by spill-in from the white matter^66,67^. FTP PET measures were calculated as standardized uptake value ratios (SUVRs) across the entire brain (calculated at the voxel-wise level). For the voxel-wise FTP PET analyses, we implemented the extended Müller–Gartner correction for partial volume effects. We applied surface-smoothing equivalent to 8-mm full width at a half maximum Gaussian kernel. ROI analyses, using the anatomic parcellation of FreeSurfer, underwent PVC using the GTM method as implemented in FreeSurfer^63^. The mean time difference between the FTP PET baseline and follow-up was 2.71 years (s.d. 0.87).

### Neuropsychological testing (in vivo dataset)

The PACC5 score^68^ was designed to be sensitive to cognitive change associated with preclinical AD. The PACC5 *z*-score is composed of the mean of the *z*-transformed scores of five neuropsychological tests: (1) the MMSE; (2) the Logical Memory Delayed Recall from the Weschler Memory Scale, Revised; (3) the Digit Symbol Substitution Test; (4) the sum of free and total scores from the Free and Cued Selective Reminding Test; and (5) the Category Fluency Test. We used the neuropsychological data closest to the imaging data. The mean difference between the baseline imaging session and the baseline neuropsychological testing session was 0.23 years (s.d. 0.45).

### Neuropathological measures (MAP dataset)

In the MAP study, brains were extracted and weighed immediately after the participants’ death and the brainstem and cerebellar hemispheres were removed. Both hemispheres and the brainstem were sectioned into 1-cm-thick coronal slabs. One hemisphere was frozen, as were select samples of the brainstem; the remaining hemisphere was fixed in 4% paraformaldehyde. Neuronal density (per mm^2^) of the LC was examined using immunohistochemistry with a monoclonal anti-tyrosine hydroxylase antibody. Paired helical filaments tau tangle density of the LC, hippocampus, EC, IT cortex and the other regions available in this dataset was examined using immunohistochemistry with a phospho-tau antibody AT8 (density per mm^2^). Neuronal and tangle density of the LC were measured bilaterally at two levels of the LC (rostral and main body) and aggregated into one total score^9,69,70^. Neocortical Aβ load was quantified as percent area occupied by Aβ, labeled with an N-terminal directed monoclonal antibody, which identifies both the 1–40 and 1–42 length Aβ fragments. The modified Bielschowsky silver quantification was used for the Braak scoring of neurofibrillary pathology and the Consortium to Establish a Registry for AD scoring of neuritic plaques. This evaluation is performed independent of clinical information, including the diagnosis^57,71^.

### Statistics and reproducibility

To test the hypothesized pathway of pathologic spreading from the LC to brain allo- and neocortical areas, we used the serial LC MRI and tau PET neuroimaging data of 77 individuals and performed voxel-wise regression analysis between inverted LC intensity_r_ and whole-brain tau accumulation (using individual FTP PET images) in MATLAB (v.R2017a, https://www.mathworks.com/products/matlab.html). All four directional models were computed between baseline and follow-up measures of LC intensity_r_ and FTP binding, with sex and age as covariates of no interest. We performed additional analyses to control for (1) CDR status; (2) neocortical PiB burden (global Aβ); and (3) the choroid plexus FTP signal (we used a two-step correction: first, we removed the effect of the choroid plexus from the FTP PET images using a general linear model (GLM), and then we performed a regression analysis between LC intensity_r_ and the corrected tau images). Additionally, to test the robustness of the voxel-wise analysis, we tested these associations also at the ROI level (GTM PVC) using robust linear regression and adjusting for age, sex, CDR and neocortical PiB burden. Furthermore, to investigate whether our hypothesized LC–allocortical tau spreading pathways were dependent of Aβ pathology, we computed a voxel-wise regression analysis between the interaction of neocortical PiB binding at baseline and LC intensity_r_ (at baseline) on whole-brain allo- and neocortical tau accumulation (at follow-up). Then, we conducted a Johnson–Neyman analysis to determine the range of Aβ values, where LC intensity_r_ was significantly associated with tau in the regions revealed in the voxel-wise analysis (*P* = 0.05; conversion to CL^72^ is provided in the results). All neuroimaging results were whole-brain-corrected for multiple comparisons utilizing a cluster-wise Monte-Carlo simulation method with 10,000 iterations to estimate the probability of false-positive clusters with a two-tailed *P* < 0.05 (3dClustSim; AFNI, https://afni.nimh.nih.gov/). To compare the different models statistically (baseline LC intensity_r_ related to follow-up whole-brain tau accumulation versus baseline whole-brain tau deposition related to follow-up LC intensity_r_), we extracted correlation coefficients of the voxels within the clusters surviving the multiple comparison using MATLAB. We tested distribution differences of these coefficients using pairwise *t*-statistics and examined the relationship between these distributions with Pearson’s correlation coefficients using R (v.4.1.3, https://www.r-project.org/).

To investigate the relationship between our hypothesized pathologic spreading pathway and cognitive performance, we performed robust linear regression analyses, using the individuals’ LC-related follow-up MTL tau values as predictors (averaged voxel FTP values (SUVR and PVC) extracted from the clusters surviving multiple comparison correction) and the PACC5 (*z*-scores) as outcome measures. We included sex, age, years of education and CDR as covariates. The analyses were conducted separately for each hemisphere tau values (left or right MTL) and for the averaged left and right MTL tau values. Afterwards, to model the relationship between this hypothesized pathway and cognitive performance approximately 3 years later, we conducted a mediation analysis with a nonparametric bootstrap approach (5,000 simulations), with baseline LC intensity_r_ as the predictor, follow-up MTL tau as the mediator and follow-up PACC5 (*z*-scores) as the outcome.

To validate the in vivo neuroimaging results in the MAP cohort, we used partial Spearman rank correlations to relate tangle density in LC to tangle density in the hippocampus, EC and IT cortex in unimpaired cognitive participants and impaired individuals (participants with MCI or AD). These analyses were adjusted for age, sex, LC neuronal density, postmortem interval and in a second step for global Aβ burden. Relationships between LC tangle density and all the other regions available in the MAP are provided in the supplementary data. Postmortem data often use staging to infer the spatiotemporal sequence of pathology, but the LC is not included in the Braak staging information in MAP (Braak stage a–c involving subcortical lesions^73^). There are also no clear cutoff points in the regional tangle density measures to delimit the stages. Thus, to understand the temporal positioning of LC tangles relative to allocortical tangle pathology, we first identified the maximum LC tangle density representative of high LC tau but low likelihood of allocortical tau accumulation (Braak stage a–c). Thus, we used all the individuals at pre-cortical Braak stage 0 for our first cutoff and extracted the maximum LC tangle density value (cutoff of 0.379 counts per mm^2^). Then, to be able to infer the staging unbiased by neocortical pathology information, we selected only the cognitive unimpaired cases with LC tangle density values below this cutoff, independent of their Braak staging and determined the cutoff points based on the mean value along with its 95% CI of the LC tangle density (mean 0.143 counts per mm^2^, 95% CI 0.049–0.238). For the allocortical tangle density, we used the same approach. We categorized individuals into low versus high groups. First, we selected the group with one Braak stage lower than the one of interest (for example, when interested in Braak stage III, we selected everyone in Braak stage II) using the available Braak staging in the MAP data. Then, we calculated the mean and 95% CI of all the cognitively unimpaired participants identified below this maximum allocortical cutoff. The probability of being in low/high tau groups was evaluated with the exact binomial test. The differences in Aβ burden across the four groups was assessed with the pairwise Welch’s *t*-test.

To study whether the LC displays similar gene expression profiles to other areas in the human brain and to understand specific biological features underlying the vulnerability of early tau pathways from the LC to the (sub)cortex, we used the AHBA and a correlation strategy to find associations between the gene expressions of LC and the rest of brain regions. Using a neurogenetic approach allows us to generate hypotheses about the neurobiological basis of the initial progression of tau through the brain. To conduct the neurogenetic analysis, we used the Desikan–Killiany 68 brain ROI atlas^74^ to conduct a surface anatomical transformation of the AHBA transcriptome. In addition, we added bilateral LC (using the samples designated as LC in the AHBA reference annotations) and the left and right hippocampus and left and right amygdala (using the FreeSurfer segmentation atlas). As in previous works^47,75–78^, to derive the transcriptome data for our custom atlas, we followed three steps for each AHBA individual and obtained a group expression matrix with the median values: (1) expression values from multiple probes were mean averaged for each gene; (2) each sample was mapped to a brain region (neocortical or subcortical) in which the samples not assigned to any region were evaluated and the samples with a distance <3 mm to any neocortical or subcortical region was assigned to it; and (3) the median genetic expression across all samples within each brain neocortical or subcortical region were computed. A group expression map was computed after calculating the median expression values of the six individual donors. The correlation analysis between the gene expression of the LC and that of the other 70 brain regions (68 neocortical regions from the Desikan–Killiany atlas, plus hippocampus and amygdala) was conducted in MATLAB. A permutation analysis was run with 10,000 permutations to test the stability of the correlation coefficient between LC and the most correlated regions. An intersection analysis defined common protein-coding genes between LC (top 5% genes) and the five most correlated brain regions (top 5% genes per region). Finally, we used GO terms^79,80^ within Metascape^81^ to conduct an enrichment analysis and to obtain GO functionalities from the LC neurogenetic results. This analysis yields a list of the main biological functions (enriched terms) shared across common protein-coding genes expressed at LC and the five most correlated brain regions. The intersection analysis was repeated as a final exploratory step, introducing a list of 75 AD-related genes^34^. We focused on intersections between the AD genes, LC and at least one of the five most correlated brain regions. To complete this analysis, we calculated the probability of finding meaningful associations. We generated 10,000 random samples of AHBA genes matching the total number of genes shared by LC and the five most correlated brain regions. Then, we calculated one-tail probabilities of finding matching AD genes and random AHBA genes.

No advance pre-determination of sample size was performed, we used all the available data of each dataset. No data were excluded from the analyses. No randomization method was used to allocate to experimental groups. For ex vivo data, individuals were assigned to the groups (unimpaired or impaired) according to their last clinical visit before autopsy^56^. Data normality and equal variance were tested, when distributions were not normal the appropriate statistical tests were used and the individual points are displayed where possible. Only when needed, individuals with missing information at some time point in specific variables were removed from specific analysis (as described in the results). Age, sex, years of education, CDR and/or Aβ (neocortical PiB binding) have been used in a stepwise manner as covariates in our linear regression analysis as they can potentially account for some variability in the outcome measures. Including these covariates increases the accuracy of the statistical models, reduces type I and type II errors and isolates the effects of the independent variables tested. Experimenters collecting MRI or behavioral data were blind to Aβ status or tau binding and experimenters collecting PET data were blind to the behavioral data and MRI results. Images were de-identified and de-faced before imaging processing.

### Reporting summary

Further information on research design is available in the Nature Portfolio Reporting Summary linked to this article.

### Supplementary information

Supplementary Information Supplementary Tables 1–9.

Reporting Summary

### Source data

Source Data Fig. 1 Data for reproducing the plot(s) in Fig. 1b. See Data Availability Statement for data related to other panels.

Source Data Fig. 2 Data for reproducing the plot(s) in Fig. 2b. See Data Availability Statement for data related to other panels.

Source Data Fig. 3 Data for reproducing the plot(s) in Fig. 3b,c. See Data Availability Statement for data related to other panels.

Source Data Extended Data Fig. 2 Data for reproducing plots in Extended Data Fig. 2.

Source Data Extended Data Fig. 3 Data for reproducing plots in Extended Data Fig. 3.

Source Data Extended Data Fig. 8 Data for reproducing the plot(s) in Extended Data Fig. 8.

Source Data Extended Data Fig. 9 Data for reproducing the plot(s) in Extended Data Fig. 9.

## Data Availability

The HABS project is committed to publicly releasing its data. Baseline data are available online at http://nmr.mgh.harvard.edu/lab/harvardagingbrain/data. Follow-up data of the HABS data, including the data used in this manuscript, will be publicly available to the research community online at http://nmr.mgh.harvard.edu/lab/harvardagingbrain/data. Data until year 5 are currently available by request, pending approval of a data request and agreement to abide by the HABS online data use agreement. We provide Source Data for figures and extended data figures. Data from the MAP are available upon request at www.radc.rush.edu. Data from the AHBA are available at https://human.brain-map.org. Source data are provided with this paper.

## Code availability

The code used in the study is generic and well documented in existing literature (GLMs and regression models). Imaging analysis was conducted using common freely available software: FreeSurfer v.6 (https://surfer.nmr.mgh.harvard.edu/), MATLAB R2017a, R package v.2022.07.1+554 (https://www.R-project.org/), 3dClustSim; AFNI, https://afni.nimh.nih.gov/), FSLeyes v.1.0.13 (FMRIB Centre, https://fsl.fmrib.ox.ac.uk/fsl/fslwiki/), SurfIce v. 6 October 2021 (https://www.nitrc.org/projects/surfice/). Specific reasonable questions can be addressed to the corresponding authors and codes will be shared on request from a qualified academic investigator.
