# Supplementary material for: Spatiotemporal patterns of locus coeruleus integrity predict cortical tau and cognition
Source: Nat Aging. 2024 Apr 25;4(5):625–37. doi: 10.1038/s43587-024-00626-y (PMC11108787; doi:10.1038/s43587-024-00626-y)
Supplement: Supplementary file 1 — Supplementary Tables 1–9. [file 43587_2024_626_MOESM1_ESM.pdf]

nature aging

Letter

https://doi.org/10.1038/s43587-024-00626-y

Spatiotemporal patterns of locus coeruleus integrity predict cortical tau and cognition

In the format provided by the authors and unedited

SUPPORTING INFORMATION

This file contains the following Supplementary Tables:

- Supplementary Table 1. Characteristics of the sample. A) In-vivo tau dataset. B) Ex-vivo MAP dataset.
- Supplementary Table 2. Summary peak maxima statistics for the MTL clusters found in the in-vivo neuroimaging analysis investigating the relationship between LC $intensity_r$ at baseline and longitudinal cortical tau accumulation.
- Supplementary Table 3. Summary peak maxima statistics for the clusters found in the in-vivo neuroimaging analysis investigating the interaction between neocortical PiB-burden (Aβ) and LC $intensity_r$ on longitudinal cortical tau accumulation.
- Supplementary Table 4. Spearman-rank partial correlation results between tangle density in LC and three regions of interest – hippocampus, entorhinal cortex (EC), and inferior temporal cortex (IT).
- Supplementary Table 5. Robust linear regression results between the LC-related tau clusters and PACC5 z-score.
- Supplementary Table 6. Robust linear regression results between the LC-related tau clusters and PACC5 z-score when including controlling for neocortical PiB-burden (Aβ).
- Supplementary Table 7. Final list of 298 genes, which was obtained when considering the intersections between LC and only one other region.
- Supplementary Table 8. Enrichment analysis results.
- Supplementary Table 9. Probability of identifying AD risk genes within the LC-MTL-limbic common genes.

Supplementary Table 1. Characteristics of the sample. A) In-vivo tau dataset. B) Exvivo MAP dataset

**A) In-vivo tau dataset**		
	**Baseline**	**Follow-up**
n (% females)	77 (65%)	
Mean age (years) (SD)	66.98 (13.09)	69.69 (7.64)
Mean years of education (SD)	15.96 (2.66)	
MMSE (score) (SD)	29.08 (1.21)	28.79 (1.17)
CDR: n = 0 | 0.5	74 | 3	71 | 6
Mean LC intensity$_r$ (SD)	1.33 (0.05)	1.30 (0.04)
Mean PACC5 (z-score) (SD)	0.37 (0.70)	0.28 (0.80)
**B) Exvivo MAP dataset**		
	**Unimpaired participants**	**Impaired participants**
n (% females)	66 (72.73%)	94 (63.83%)
Mean age of death (years) (SD)	87.23 (5.93)	89.70 (5.09)
Mean years of education (SD)	13.91 (2.58)	15.29 (2.59)
Mean MMSE (score) (SD)	27.90 (1.81)	20.89 (7.90)
Mean postmortem interval (hours) (SD)	2.33 (3.09)	7.42 (4.47)
Aβ (%) (SD)	2.33 (2.80)	5.46 (4.98)
Mean tangle density HIPP (SD)	9.89 (11.83)	19.59 (16.42)
Mean tangle density EC (SD)	12.51 (13.11)	17.97 (12.34)
Mean tangle density IT (SD)	2.32 (6.99)	8.07 (11.32)
Mean tangle density LC (SD)	1.39 (1.58)	2.77 (2.64)
Mean neuron density LC (SD)	48.7 (17.4)	44.43 (19.13)

Missing data in-vivo dataset: baseline and follow-up MMSE: 3 participants; follow-up PACC5: 3 participants. **Missing data ex-vivo dataset:** MMSE, 1 unimpaired participant, and 7 impaired participants had missing values; tangle density of the EC: 1 unimpaired participant. **Abbreviations:** Aβ = beta-amyloid, CDR = Clinical Dementia Rating (CDR =0, no impairment; CDR = 0.5, very mild dementia); EC = entorhinal cortex; HIPP = hippocampus; IT = inferior temporal; LC = locus coeruleus; MMSE = Mini-Mental State Examination; n = total number of individuals; SD = standard deviation. **Notes:** Neuronal density (counts/mm^2) of the LC was examined using immunohistochemistry with a monoclonal anti-tyrosine hydroxylase antibody and paired helical filaments tau tangle density of the LC, hippocampus, entorhinal cortex, and inferior temporal cortex was examined using immunohistochemistry with an anti-paired helical filaments tau antibody AT8 (counts/mm^2).

Return to top

Supplementary Table 2. Summary peak maxima statistics for the MTL clusters found in the in-vivo neuroimaging analysis investigating the relationship between LC intensity$_r$ at baseline and longitudinal cortical tau accumulation. We used whole-brain voxel-wise level GLM analysis (one-sided, p-value<0.05 cluster-corrected for multiple comparisons). Abbreviations: CDR= Clinical Dementia Rating; L = Left; MTL = medial temporal lobe; R = right.

**Covariates**	**Region**	**z-statistic**	**p-value**
Sex and age	R MTL	2.78	0.005
	L MTL	2.94	0.003
Sex, age, CDR, and Aβ	R MTL	2.72	0.007
	L MTL	2.75	0.006
Choroid plexus signal residualized Sex and age	R MTL	2.65	0.008
	L MTL	2.05	0.040
Choroid plexus signal residualized Sex, age, CDR, and Aβ	R MTL	2.29	0.022
	L MTL	1.74	0.049

Return to top

Supplementary Table 3. Summary peak maxima statistics for the clusters found in the in-vivo neuroimaging analysis investigating the interaction between neocortical PiB-burden (Aβ) and LC intensity$_{r}$ on longitudinal cortical tau accumulation. We used whole-brain voxel-wise level GLM analysis (one-sided, p-value<0.05 cluster-corrected for multiple comparisons). Abbreviations: CDR= Clinical Dementia Rating; L = Left; MTL = medial temporal lobe; R = right.

Covariates	Region	z-statistic	p-value
Sex and age	L MTL	3.36	0.002
	R MTL	3.99	0.001
	R occipital cortex	3.64	0.001
Sex age and CDR	L MTL	2.72	0.007
	R MTL	3.23	0.002
	R occipital cortex	3.38	0.001
Choroid plexus signal residualized, sex, age	L MTL	3.28	0.002
	R MTL	4.21	0.001
	R occipital cortex	3.45	0.001
Choroid plexus signal residualized sex, age and CDR	L MTL	2.63	0.009
	R MTL	3.31	0.001
	R occipital cortex	3.43	0.006

Return to top

Supplementary Table 4. Spearman-rank partial correlation results between tangle density in LC and three regions of interest – hippocampus, entorhinal cortex (EC), and inferior temporal cortex (IT). Analyses were done separately for unimpaired and impaired (MCI/AD) individuals, adjusted for sex, age, postmortem interval, and global Aβ score (two-sided, $p<0.05$, uncorrected for multiple comparisons).

	**RHO**	**p-value**	**n**
Unimpaired individuals			
Hippocampus	0.57	7.027e-05 ***	47
EC	0.64	5.389e-06 ***	46
IT	0.38	0.0138 *	46
Impaired individuals			
Hippocampus	0.47	2.194e-05 ***	79
EC	0.48	1.408e-05 ***	78
IT	0.71	2.551e-12 ***	78

Legend: p-value < 0.05 = *, p-value < 0.001 = **, p-value < 0.0001 = ***.

Return to top

Supplementary Table 5. Robust linear regression results between the LC-related tau clusters and PACC5 z-score. Regression analyses were two-sided, p<0.05, uncorrected for multiple comparisons.

**Right LC-MTL cluster**		**Estimate**	**Std. error**	**t-value**	**p-value**
	Right LC-MTL	-2.01	0.40	-4.99	4.61e-06***
	Sex	0.40	0.14	2.82	0.01*
	Years education	0.04	0.03	1.75	0.08
	Age	-0.01	0.01	-1.41	0.16
	CDR	-0.83	0.69	-1.21	0.23
**Left LC-MTL cluster**		Estimate	Std. error	t-value	p-value
	Left LC-MTL	-2.14	0.48	-4.42	3.57e-05***
	Sex	0.39	0.15	2.64	0.01*
	Years education	0.05	0.03	1.98	0.05*
	Age	-0.02	0.01	-2.04	0.05*
	CDR	-0.47	0.71	-0.65	0.50

Legend: p-value < 0.05 = *, p-value < 0.001 = **, p-value < 0.0001 = ***.

Return to top

Supplementary Table 6. Robust linear regression results between the LC-related tau clusters and PACC5 z-score when including controlling for neocortical PiB-burden (Aβ). Regression analyses were two-sided, p<0.05, uncorrected for multiple comparisons.

**Right LC-MTL cluster**		**Estimate**	**Std. error**	**t-value**	**p-value**
	Right LC-MTL	-2.21	0.42	-5.33	1.62e-06***
	Sex	0.42	0.15	2.89	0.01*
	Years education	0.04	0.03	1.63	0.10
	Age	-0.01	0.01	-1.47	0.14
	CDR	-0.98	0.72	-1.37	0.17
	Aβ	0.42	0.39	1.07	0.31
**Left LC-MTL cluster**		**Estimate**	**Std. error**	**t-value**	**p-value**
	Left LC-MTL	-2.44	0.55	-4.44	4.02e-05***
	Sex	0.41	0.16	2.56	0.01*
	Years education	0.05	0.03	1.80	0.07
	Age	-0.02	0.01	-2.03	0.05*
	CDR	-0.71	0.78	-0.90	0.36
	Aβ	0.40	0.44	0.89	0.38

Legend: p-value < 0.05 = *, p-value < 0.001 = **, p-value < 0.0001 = ***.

Return to top

Supplementary Table 7. Final list of 298 genes, which was obtained when considering the intersections between LC and only one other region.

**LC ∩ mOFC**	**LC ∩ hippocampus**	**LC ∩ insula**	**LC ∩ amygdala**	**LC ∩ rACC**
N=58	N=62	N=56	N=77	N=45
ANKRD55	APOL6	ACLY	ABHD3	ACOT12
ATBF1	ATL1	AKR1C1	AK3	AK7
B4GALT7	ATP1B1	AP3M1	AKIRIN1	ARSD
BHMT2	C12orf60	BRCC3	BAG3	AVEN
C22orf36	C14orf50	C17ORF106	C10orf88	BCL7C
C7orf73	C1orf161	C1QL4	C15orf41	BRUNOL6
CALY	CALCA	C20orf194	C17orf71	C16orf82
CCNG1	CDS2	C2orf67	C2orf29	C19orf31
CD55	CHAC2	CCDC127	C5orf28	C3orf67
CDC25A	CHAT	CDC42	C9orf61	CALB2
CDNF	CHGB	CDV3	CAPZA1	CALCR
CRNDE	CHORDC1	CEP70	CBLB	CCR7
CXorf30	CISD2	CHRFAM7A	CD164	DPYSL3
CYB5R1	CLCN3	CKAP4	CDC16	EBPL
DMRT1	COPB2	CPAMD8	CSNK1G3	ECEL1
FAM114A2	DAP3	DRD2	CYP51A1	EPHX2
FAM133A	EXOC1	EEF1E1	DAAM1	FLJ30901
FAM173B	FAM136A	ERP29	EPDR1	GALR2
FAM27A	GATA6	FGF1	EPS8	GCNT2
FAM82B	GCAT	FXR1	FARSB	GDPD2
GFPT1	HSPA4	GBE1	FTSJD1	GSTP1
GPR68	HUWE1	GPN1	FXN	HSD17B7
GRINL1A	IGFBPL1	GRB10	GALNT13	HSP90AB1
HIBCH	IMPAD1	GRN	GDF11	IRS4
HSD17B10	ITGA4	GTF2H3	GJB7	KLHDC10
HSPA13	KCNE1L	HSPB1	GNAI3	KRT18P26
IFIT5	KIAA1211	INTS4	GOLPH3L	LOC400750
IMPACT	KIF21A	KANK4	INSIG1	ME2
INS	KLHDC8A	KCTD10	IP6K2	NXNL2
KBTBD10	LARP1	KCTD20	IRX3	PCDH21
LSS	LIAS	KRTAP20-1	LHX9	PFDN2
MAPRE1	LOC100130054	L1TD1	LOC550643	PMCHL2
MMEL1	LOC100133008	LPIN1	LOC729852	POFUT2
MPZL1	LRRC16B	MRPS33	MAGT1	PTS
NANOS1	MAP1B	NEK7	MAMLD1	RPH3AL
NINL	NDUFAF2	OR6C76	MGAT2	SCEL
OLFM2	OPA1	PAFAH2	MRPL13	SGSM1
OSBPL9	PODXL2	PCYOX1L	MUT	SMAD9
PFDN4	PRKACA	PFN4	OR6C65	SPRYD4
PGAM2	PRLHR	PHACTR2	P4HA1	SRPX
PHF11	RAB39	POM121C	PDCD5	SULF1
PIPOX	RAB3GAP2	PTDSS1	PDE11A	TRMT112
PODNL1	RAB9B	PTGES3	PELI2	UBR1
PPAT	RFK	RAB30	PIGP	YKT6
REC8	RGS2	RBMS1	PIH1D2	ZMYND12
RGS3	RHBDD3	RNF152	POMC	

RIT1	RHEB	S100A10	POMP	
SGMS2	RNF128	SCN7A	PRDX6	
SHOX2	RSPO4	SERPINB8	PTGFR	
SLC41A2	SCOC	SLC16A3	RAB10	
SLC9A7	SLC46A1	SPOCK3	RAB21	
STK32A	SMARCA1	STEAP3	RAB3B	
STT3A	STRA13	TCEAL7	RAN	
SYT9	TEX15	TMEM63C	RAP1B	
TPST1	TMEM182	TP53I3	RHOQ	
VWA5A	TPH2	ZNF558	RPAP3	
WRN	TRIM61		SHC1	
ZNF568	TTC1		SLC25A46	
	VEPH1		STC1	
	WARS		STX17	
	WNT11		THAP9	
	ZNF676		TMEM167A	
			TMEM170A	
			TMEM19	
			TMEM27	
			TMEM38B	
			TMEM97	
			TRAM1	
			UPRT	
			VBP1	
			WDR48	
			WWTR1	
			XPO4	
			ZC3HAV1	
			ZMPSTE24	
			ZNF10	
			ZNF569	

Return to top

Supplementary Table 8. Enrichment analysis results. Protein-coding genes associated with the main gene ontology functionality: regulation of protein transport.

**Regulation of protein transport (n=34)**			
CDC42	RPH3AL	RAB21	MAPRE1
CYP51A1	BAG3	CALY	GRB10
DRD2	HUWE1	MAP1B	S100A10
HSP90AB1	ERP29	WWTR1	ZMPSTE24
INS	RHBDD3	CALCA	
INSIG1	GOLPH3L	FGF1	
PRKACA	EXOC1	HSPB1	
RAN	XPO4	RHOQ	
GPR68	NDUFAF2	CBLB	
PDCD5	SYT9	ITGA4	

Return to top

Supplementary Table 9. Probability of identifying AD risk genes within the LC-MTL-limbic common genes. A random permutation analysis (10,000 iterations) and 1-tail probabilities were calculated.

Number of matching genes	Probability
0	18.1 %
1	18.5 %
2	9.5 %
3	3.0 %
4	0.7 %
5	0.2 %
6	< 0.01 %

Return to top
