# Supplementary material for: Spatiotemporal patterns of locus coeruleus integrity predict cortical tau and cognition
Source: Nat Aging. 2024 Apr 25;4(5):625–37. doi: 10.1038/s43587-024-00626-y (PMC11108787; doi:10.1038/s43587-024-00626-y)
Supplement: Supplementary file 2 — Reporting Summary [file 43587_2024_626_MOESM2_ESM.pdf]

nature portfolio

Corresponding author(s): Jorge Sepulcre and Heidi I. L. Jacobs

Last updated by author(s): Feb 21, 2024

Reporting Summary

Nature Portfolio wishes to improve the reproducibility of the work that we publish. This form provides structure for consistency and transparency in reporting. For further information on Nature Portfolio policies, see our Editorial Policies and the Editorial Policy Checklist.

Statistics

For all statistical analyses, confirm that the following items are present in the figure legend, table legend, main text, or Methods section.

n/a	Confirmed	
☐	☒	The exact sample size (*n*) for each experimental group/condition, given as a discrete number and unit of measurement
☐	☒	A statement on whether measurements were taken from distinct samples or whether the same sample was measured repeatedly
☐	☒	The statistical test(s) used AND whether they are one- or two-sided<br>*Only common tests should be described solely by name; describe more complex techniques in the Methods section.*
☐	☒	A description of all covariates tested
☐	☒	A description of any assumptions or corrections, such as tests of normality and adjustment for multiple comparisons
☐	☒	A full description of the statistical parameters including central tendency (e.g. means) or other basic estimates (e.g. regression coefficient) AND variation (e.g. standard deviation) or associated estimates of uncertainty (e.g. confidence intervals)
☐	☒	For null hypothesis testing, the test statistic (e.g. *F*, *t*, *r*) with confidence intervals, effect sizes, degrees of freedom and *P* value noted<br>*Give P values as exact values whenever suitable.*
☒	☐	For Bayesian analysis, information on the choice of priors and Markov chain Monte Carlo settings
☒	☐	For hierarchical and complex designs, identification of the appropriate level for tests and full reporting of outcomes
☐	☒	Estimates of effect sizes (e.g. Cohen's *d*, Pearson's *r*), indicating how they were calculated

Our web collection on statistics for biologists contains articles on many of the points above.

Software and code

Policy information about availability of computer code

Data collection	No software was used for data collection.
Data analysis	FreeSurfer v6 (https://surfer.nmr.mgh.harvard.edu/)<br>MATLAB R2017a<br>R package version 2022.07.1+554 (https://www.R-project.org/)<br>3dClustSim; AFNI: https://afni.nimh.nih.gov/).<br>FSLeyes version 1.0.13 (FMRIB centre, Oxford, UK; https://fsl.fmrib.ox.ac.uk/fsl/fslwiki/)<br>Surf Ice version October 6, 2021 (https://www.nitrc.org/projects/surfice/)

For manuscripts utilizing custom algorithms or software that are central to the research but not yet described in published literature, software must be made available to editors and reviewers. We strongly encourage code deposition in a community repository (e.g. GitHub). See the Nature Portfolio guidelines for submitting code & software for further information.

Data

Policy information about availability of data

All manuscripts must include a data availability statement. This statement should provide the following information, where applicable:

- Accession codes, unique identifiers, or web links for publicly available datasets
- A description of any restrictions on data availability
- For clinical datasets or third party data, please ensure that the statement adheres to our policy

The HABS project is committed to publicly releasing its data. Baseline data are already available online at http://nmr.mgh.harvard.edu/lab/harvardagingbrain/data. Follow-up data of the HABS data, including the data used in this manuscript, will be publicly to the research community. Data until year 5 are currently available by request, pending approval of a data request and agreement to abide by the HABS online data use agreement. Data from the MAP are available upon request at www.radc.rush.edu. Data from the AHBA are available upon request at https://human.brain-map.org.

Research involving human participants, their data, or biological material

Policy information about studies with human participants or human data. See also policy information about sex, gender (identity/presentation), and sexual orientation and race, ethnicity and racism.

Reporting on sex and gender	The covariable "sex" was used in this work as a control variable within statistical analysis. Individuals' sex information was used according to the information available in the corresponding database (HABS dataset or MAP dataset).<br>HABS dataset: n=77, 50 females (65%)<br>MAP dataset: n=66, 48 females (72.73%)
Reporting on race, ethnicity, or other socially relevant groupings	*Please specify the socially constructed or socially relevant categorization variable(s) used in your manuscript and explain why they were used. Please note that such variables should not be used as proxies for other socially constructed/relevant variables (for example, race or ethnicity should not be used as a proxy for socioeconomic status).*<br>*Provide clear definitions of the relevant terms used, how they were provided (by the participants/respondents, the researchers, or third parties), and the method(s) used to classify people into the different categories (e.g. self-report, census or administrative data, social media data, etc.)*<br>*Please provide details about how you controlled for confounding variables in your analyses.*
Population characteristics	Data used in this work comes from: (i) HABS, (ii) MAP study. Demographic information for these samples has been fully described within the manuscript and in Table 1.<br>Recruitment<br>HABS dataset (in-vivo)<br>n = 77, 50 females (65%).<br>Mean age (years) (SD): baseline: 66.98 (13.09) I follow-up 69.69 (7.64)<br>Mean years of education (SD): 15.96 (2.66)<br>MMSE (score) (SD): baseline 29.08 (1.21) I follow-up 28.79 (1.17)<br>CDR n = 0: baseline 74 I follow-u: 71<br>CDR n= 0.5: baseline 3 I follow-up 6<br>Mean LC intensityr (SD): baseline 1.33 (0.05) I follow-up 1.30 (0.04)<br>Mean PACCS (z-score) (SD): baseline 0.37 (0.70) I follow-up 0.28 (0.80)<br>At baseline, mean neocortical amyloid-beta = 1.261 DVR (SD =0.21; amyloid-beta positive: 10 participants, amyloid-beta negative 62 participants; follow-up data from 3 participants was used for calculating the baseline mean, and 2 participants had missing PiB-values in baseline or follow-up).<br>MAP dataset (ex-vivo)<br>Unimpaired participants /// impaired partiicpants<br>n (% females) 66 (72.73%) /// 94 (63.83%)<br>Mean age of death (years) (SD) 87.23 (5.93) /// 89.70 (5.09)<br>Mean years of education (SD) 13.91 (2.58) ///15.29 (2.59)<br>Mean MMSE (score) (SD) 27.90 (1.81) ///20.89 (7.90)<br>Mean postmortem interval (hours) (SD) 2.33 (3.09) /// 7.42 (4.47)<br>Amyloid-beta (%) (SD) 2.33 (2.80) /// 5.46 (4.98)<br>Mean tangle density HIPP (SD) 9.89 (11.83) /// 19.59 (16.42)<br>Mean tangle density EC (SD) 12.51 (13.11) /// 17.97 (12.34)<br>Mean tangle density IT (SD) 2.32 (6.99) /// 8.07 (11.32)<br>Mean tangle density LC (SD) 1.39 (1.58) /// 2.77 (2.64)<br>Mean neuron density LC (SD) 48.7 (17.4) /// 44.43 (19.13)<br>Missing data in-vivo dataset: baseline and follow-up MMSE: 3 participants; follow-up PACCS: 3 participants. Missing data exvivo<br>dataset: MMSE, 1 unimpaired participant, and 7 impaired participants had missing values; tangle density of the EC: 1 unimpaired participant
Recruitment	In-vivo dataset: the behavioral and imaging data (fMRI and PET) used in this work comes from the Harvard Aging Brain Study (HABS) and the affiliated LOCUST-study.<br>REFERENCE: Jacobs, H. I. L. et al. In vivo and neuropathology data support locus coeruleus integrity as indicator of Alzheimer's disease pathology and cognitive decline. Sci. Transl. Med. 13, eabj2511 (2021).<br>Ex-vivo dataset: this sample is part of the from the Rush Memory and Aging Project.<br>REFERENCES:<br>Bennett, D. A. et al. Overview and Findings from the Rush Memory and Aging Project. Curr. Alzheimer Res. 9, 646-663.

Bennett, D. A. et al. Religious Orders Study and Rush Memory and Aging Project. J. Alzheimers Dis. JAD 64, S161—S189 (2018)).
Genetic data: we used the Allen Human Brain Atlas (AHBA), which is a transcriptional atlas of the adult human brain derived from histological analysis and microarray profiling.
REFERENCE: Hawrylycz, M. J. et al. An anatomically comprehensive atlas of the adult human brain transcriptome. Nature 489, 391-399 (2012

Ethics oversight	Institutional review board approval was secured at the participating sites.<br><br>The Partners Human Research Committee approved the research protocols of Massachusetts General Hospital. All participants provided written informed consent received monetary compensation after each visita<br>The HABS project is committed to publicly releasing its data. Baseline data are already available online at http://nmr.mgh.harvard.edu/lab/harvardagingbrain/data. Follow-up data of the HABS data, including the data used in this manuscript, will be publicly to the research community. Data until year 5 are currently available by request, pending approval of a data request and agreement to abide by the HABS online data use agreement. Data from the MAP are available upon request at www.radc.rush.edu.

Note that full information on the approval of the study protocol must also be provided in the manuscript.

Field-specific reporting

Please select the one below that is the best fit for your research. If you are not sure, read the appropriate sections before making your selection.

☒ Life sciences ☐ Behavioural & social sciences ☐ Ecological, evolutionary & environmental sciences

For a reference copy of the document with all sections, see nature.com/documents/nr-reporting-summary-flat.pdf

Life sciences study design

All studies must disclose on these points even when the disclosure is negative.

Sample size	Sample size was determined using the available data for our research questions from each dataset.<br>In-vivo sample = 77 individuals<br>Ex-vivo sample = 160 individuals<br>AHBA genetic data: genetic expression of 20,737 protein-coding genes extracted from 58,692 measurements from 3,702 brain samples. Data was originally obtained from six donors.
Data exclusions	No data exclusions.<br>Missing data in-vivo dataset: baseline and follow-up MMSE: 3 participants; follow-up PACC5: 3 participants. Missing data ex-vivo dataset: MMSE, 1 unimpaired participant, and 7 impaired participants had missing values; tangle density of the EC: 1 unimpaired participant.
Replication	The data used in this work is unique. Replication analysis have not been conducted because there is a lack of available cohorts with the same data availability.
Randomization	In-vivo sample consisting of 77 individuals not allocated into groups.<br>Ex-vivo sample consisting of 160 individuals: 66 individuals with normal cognition and 94 MCI or AD individuals at their last clinical visit prior to autopsy (reference: Bennett, D. A. et al. Natural history of mild cognitive impairment in older persons. Neurology 59, 198-205 (2022)).<br>AHBA genetic expression was not allocated into groups.
Blinding	Data collection was performed blind to the condition of the experiments. Images are de-identified and de-faced.

Reporting for specific materials, systems and methods

We require information from authors about some types of materials, experimental systems and methods used in many studies. Here, indicate whether each material, system or method listed is relevant to your study. If you are not sure if a list item applies to your research, read the appropriate section before selecting a response.

Materials & experimental systems

n/a	Involved in the study	
☒	☐	Antibodies
☒	☐	Eukaryotic cell lines
☒	☐	Palaeontology and archaeology
☒	☐	Animals and other organisms
☒	☐	Clinical data
☒	☐	Dual use research of concern
☒	☐	Plants

Methods

n/a	Involved in the study	
☒	☐	ChIP-seq
☒	☐	Flow cytometry
☐	☒	MRI-based neuroimaging

Plants

Seed stocks

Report on the source of all seed stocks or other plant material used. If applicable, state the seed stock centre and catalogue number. If plant specimens were collected from the field, describe the collection location, date and sampling procedures.

Novel plant genotypes

Describe the methods by which all novel plant genotypes were produced. This includes those generated by transgenic approaches, gene editing, chemical/radiation-based mutagenesis and hybridization. For transgenic lines, describe the transformation method, the number of independent lines analyzed and the generation upon which experiments were performed. For gene-edited lines, describe the editor used, the endogenous sequence targeted for editing, the targeting guide RNA sequence (if applicable) and how the editor was applied.

Authentication

Describe any authentication procedures for each seed stock used or novel genotype generated. Describe any experiments used to assess the effect of a mutation and, where applicable, how potential secondary effects (e.g. second site T-DNA insertions, mosiacism, off-target gene editing) were examined.

Magnetic resonance imaging

Experimental design

Design type

Structural

Design specifications

Two structural scans per participant.

Behavioral performance measures

Behavioral measures were not acquired during MRI scanning.

Acquisition

Imaging type(s)

Structural

Field strength

3T

Sequence & imaging parameters

MRI studies were performed at the Massachusetts General Hospital, Athinoula A. Martinos Center for Biomedical Imaging, on a 3T imaging system (TRIM Trio, Siemens). Participants were reinforced to stay still, and a short acquisition time was used to minimize motion (for more details, see 9). The MRI protocol included a structural 3D Tl-weighted volumetric magnetization—prepared rapid acquisition gradient-echo images (repetition time = 2300 ms, echo time = 2.95 ms, inversion time = 900 ms, flip angle = 9°, and 1.05 mm by 1.05 mm by 1.20 mm resolution) and an optimized MRI acquisition for locating the LC (a two-dimensional (2D) Tl-weighted turbo-spin-echo sequence with additional magnetization transfer contrast; repetition time = 743 ms, echo time = 16 ms, flip angle = 180°, six slices, four online averages, 0.4 mm by 0.4 mm by 3.00 mm resolution, and acquisition time = 3 min and 22 s).

Area of acquisition

Whole brain and partial field of view (brainstem for the LC)

Diffusion MRI ☐ Used ☒ Not used

Preprocessing

Preprocessing software

FreeSurfer version 6.0.0

Normalization

For all T1-images, the automated reconstruction protocol of FreeSurfer version 6.0.0 was performed as described in references listed below:
(i) automated segmentation, (ii) intensity normalization, (iii) skull stripping, (iv) separating left and right hemispheres, (v) excluding brainstem and cerebellum, (vi) correcting topology defects, (vii) defining the borders between gray matter, white matter, and cerebrospinal fluid, (viii) parcellating cortical and subcortical areas, (ix) visually inspecting images and, if necessary, editing them.
References:
Jacobs, H. I. L. et al. In vivo and neuropathology data support locus coeruleus integrity as indicator of Alzheimer's disease pathology and cognitive decline. Sci. Transl. Med. 13, eabj2511 (2021).
A. M. Dale, B. Fischl, M. I. Sereno, Cortical surface-based analysis. I. Segmentation and surface reconstruction. Neurolmage 9, 179-194 (1999).
Areas of interest (LC and reference region, pontine tegmentum) were registered to each individual using a combination of high-dimensional diffeomorphic with rigid-body registrations. Each slice containing the LC area was normalized to the pontine tegmentum. LC signal intensity (an indicator of LC integrity) was quantified as the mean intensity from five contiguous voxels with the highest values within LC regions of interest following 30 search iterations. Reference: Jacobs, H. I. L. et al. In vivo and neuropathology data support locus coeruleus integrity as indicator of Alzheimer's disease pathology and cognitive decline. Sci. Transl. Med. 13, eabj2511 (2021).

Normalization template

LC scans were in native resolution, not normalized. Integration MRI-PET: MNI space. Resolution of the images was 2 mm.

Noise and artifact removal

We used the automated reconstruction protocol of FreeSurfer version 6.0.0 (see above).

Volume censoring	We do not use volume measures.

Statistical modeling & inference

Model type and settings	General Linear Models (GLM)
Effect(s) tested	To investigate the spatiotemporal relationships between LC intensityr and tau accumulation, we performed voxel-wise regression analysis between inverted LC intensityr and whole-brain tau accumulation in Matlab (version R2017a, Natick, Massachusetts: The MathWorks Inc. https://www.mathworks.com/products/matlab.html). All four directional models were computed between baseline and follow-up measures of LC intensityr and FTP-binding, with sex and age as covariates of no interest. We performed the following additional analysis to control for (i) CDR status, (ii) global amyloid-beta, and (iii) the choroid plexus FTP-signal (we used a two-step correction: first, we removed the effect of choroid plexus from the FTP-PET images using a GLM, and then we did the regression analysis between LC intensityr and the corrected tau images).

Specify type of analysis: ☒ Whole brain ☐ ROI-based ☐ Both

Statistic type for inference<br>(See Eklund et al. 2016)	Cluster-wise. Monte Carlo simulation method, with 10,000 iterations to estimate the probability of false-positive clusters with a two-tailed p-value<0.05 (3dClustSim; AFNI: https://afni.nimh.nih.gov/).
Correction	Neuroimaging analysis were corrected for multiple comparisons using a cluster-wise Monte Carlo simulation method, with 10,000 iterations to estimate the probability of false-positive clusters with a two-tailed p-value<0.05 (3dClustSim; AFNI: https://afni.nimh.nih.gov/).

Models & analysis

n/a	Involved in the study
☒	☐ Functional and/or effective connectivity
☒	☐ Graph analysis
☒	☐ Multivariate modeling or predictive analysis
